# Recrudescence Mechanisms and Gene Expression Profile of the Reproductive Tracts from Chickens during the Molting Period

**DOI:** 10.1371/journal.pone.0076784

**Published:** 2013-10-01

**Authors:** Wooyoung Jeong, Whasun Lim, Suzie E. Ahn, Chul-Hong Lim, Jin-Young Lee, Seung-Min Bae, Jinyoung Kim, Fuller W. Bazer, Gwonhwa Song

**Affiliations:** 1 Department of Animal Biotechnology, Seoul National University, Gwanak-gu, Seoul, Republic of Korea; 2 Department of Animal Resources Science, Dankook University, Cheonan, Republic of Korea; 3 Center for Animal Biotechnology and Genomics and Department of Animal Science, Texas A&M University, College Station, Texas, United States of America; 4 Division of Biotechnology, College of Life Sciences and Biotechnology, Korea University, Seoul, Republic of Korea; University of Quebec at Trois-Rivieres, Canada

## Abstract

The reproductive system of chickens undergoes dynamic morphological and functional tissue remodeling during the molting period. The present study identified global gene expression profiles following oviductal tissue regression and regeneration in laying hens in which molting was induced by feeding high levels of zinc in the diet. During the molting and recrudescence processes, progressive morphological and physiological changes included regression and re-growth of reproductive organs and fluctuations in concentrations of testosterone, progesterone, estradiol and corticosterone in blood. The cDNA microarray analysis of oviductal tissues revealed the biological significance of gene expression-based modulation in oviductal tissue during its remodeling. Based on the gene expression profiles, expression patterns of selected genes such as, *TF*, *ANGPTL3*, *p20K*, *PTN*, *AvBD11* and *SERPINB3* exhibited similar patterns in expression with gradual decreases during regression of the oviduct and sequential increases during resurrection of the functional oviduct. Also, miR-1689* inhibited expression of *Sp1*, while miR-17-3p, miR-22* and *miR-1764* inhibited expression of *STAT1.* Similarly, chicken *miR-1562* and *miR-138* reduced the expression of *ANGPTL3* and *p20K*, respectively. These results suggest that these differentially regulated genes are closely correlated with the molecular mechanism(s) for development and tissue remodeling of the avian female reproductive tract, and that miRNA-mediated regulation of key genes likely contributes to remodeling of the avian reproductive tract by controlling expression of those genes post-transcriptionally. The discovered global gene profiles provide new molecular candidates responsible for regulating morphological and functional recrudescence of the avian reproductive tract, and provide novel insights into understanding the remodeling process at the genomic and epigenomic levels.

## Introduction

The chicken has unique features for basic studies of developmental processes such as organogenesis, so it is an excellent animal model for studies of vertebrate developmental biology. Under natural condition, domestic laying hens start to lay eggs after reaching sexual maturity at about 5 months of age, and the laying hen then produces an egg on 90% or more of the days in her first year of laying eggs [1]. As the hen ages, the egg production rate progressively decreases and the hen naturally undergoes annual period of molting at the end of each laying cycle [2]. Molting is a phenomenon that includes renewal of old feathers with new feathers and an associated complete remodeling of the reproductive system [2], [3]. During this process, the reproductive organs undergo regression and rejuvenation, and then recovery in preparation for a new egg laying cycle [2]. Artificially induced molting is an effective tool in commercial poultry farming for improving the rate of egg production and quality of eggs as it renews the hen's laying cycle [4], [5]. The removal of feed has been used most frequently in commercial poultry farms to induced molting in hens; however, this practice has come under criticism from animal welfare advocacy groups [6]. Furthermore, molting through feed withdrawal leads to greater susceptibility of hens to salmonella infection as compared to that for laying hens[7], [8]. Therefore, several alternative methods for inducing molting without starvation have been developed. These include feeding diets deficient in an essential nutrient or modifying the diet by feeding low levels of calcium or high levels of zinc [9]–[12]. Among these, the practice of feeding a diet high in zinc has received the most attention as it is easy to practice, post-molt performance of laying hens is acceptable and there is not an increase in susceptibility to infection by *Salmonella enteritidis* [13]–[15].

The majority of previous studies investigating molting process and associated changes in the chicken oviduct have been comparative studies of physiological changes focused on phenomena only during the period of regression of the oviduct. Therefore, relatively little is known about mechanisms regulating remodeling of the oviduct after cessation of egg production during the molting process or recrudescence of the oviduct following the molting period. Moreover, little is known about genome-based mechanisms responsible for the regenerative ability of the reproductive tract in laying hens following molting and tissue remodeling. Therefore, the present study was designed to discover novel genes and pathways underlying molecular mechanisms(s) for tissue remodeling of the oviduct of laying hens following a high zinc diet-induced molting period. A better appreciation of reproductive tissue remodeling is crucial to understanding the overall genetic and molecular mechanism(s) for both regression and regeneration of the avian female reproductive organs. Actually, most female reproductive organs undergo repetitive morphological changes according to programmed processes associated with menstrual and estrous cycles and molting process involving ovarian and reproductive tract tissues. For example, reproductive organs in women, especially uterine endometrium, undergoes dynamic tissue remodeling during some 400 menstrual cycles between puberty and menopause [16], [17]. This repetitive phenotypic change is also accompanied by dramatic changes in gene expression profiles [18].

The aims of this study were to: 1) investigate the physiological changes and gene expression profiles in reproductive organs of laying hens during molting; 2) identify novel genes and their interactions related to reproductive tissue remodeling; and 3) determine epigenetic mechanisms affecting the reproductive tract of laying hens during regression, remodeling and recrudescence associated with the period of molting. Along with completion of sequencing of the chicken genome, many applications of chicken DNA microarrays have led to massive increase in the discovery of genes whose expression is stimulated by estrogen, differential genes expression in the oviduct between immature versus mature hens, and between 3 h versus 20 h post-ovulation [19]–[21]. In the present study, we induced molting and changes in oviductal status by feeding high levels of zinc in the diet and assessed its suitability for investigations of molecular mechanisms controlling pre-molting and post-molting processes. Using cDNA microarray analysis, we now report large-scale gene expression profiles for the oviducts of laying hens during the molting and recrudescence periods. We also determined spatio-temporal specific mRNA expression patterns and validated chicken microRNAs (miRNAs) regulating these genes post-transcriptionally. Our approach contributes to the development of novel insights into degeneration and regeneration mechanisms of female reproductive organ at the molecular level.

## Results

### Morphological Changes during Induced Molting and Oviduct Recrudescence

**Figure 1:**
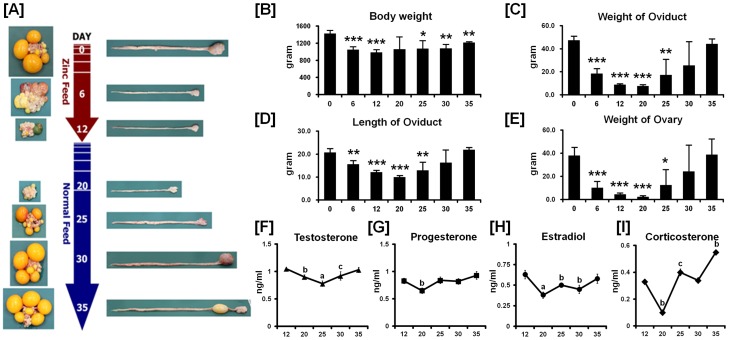
Establishment of an *in vivo* experimental model of regression and recrudescence of the chicken oviduct. (A) Experimental protocol. The numbers in the red arrows indicate high-zinc feeding days as hens gradually lost the function of the oviduct. The numbers in the blue arrows indicate days of feeding a normal commercial diet after complete cessation of egg production (day 12), and the hens begin to regain function of the oviduct. (B-E) Changes in body weight (B), oviduct weight (C), oviduct length (D) and ovarian weight (E) of hens at different days during the molting and recrudescence periods induced by feeding a high zinc diet followed by feeding a recrudescence normal commercial diet. The graphs show the mean of weights or lengths for each sample obtained on each day of the study (mean±SEM, n = 5). The asterisks denote effects that were significant (***p<0.001, ** *p<*0.01, or * *p<*0.05). (F-I) Profiles of concentrations of testosterone (F), progesterone (G), estradiol (H) and corticosterone (I) in serum from different days of the molting and recrudescence periods. Each bar represents the mean±SEM for three independent experiments. Different letters of the alphabet above the lines indicate significant differences (^a^ *p<*0.001, ^b^ *p<*0.01, or ^c^ *p<*0.05).

We identified dramatic morphological changes in oviducts and ovaries at different days during the induced molting and recrudescence periods (Figure 1A). The regression of oviducts and ovarian follicles at day 6 and day 12 (high zinc feeding days) was followed by sequentially recrudescence by feeding a normal diet after complete cessation of egg production at day 12. In order to determine morphological changes during induced molting and recrudescence, we analyzed body weight, weight and length of the oviduct, and weight of the ovaries. The average body weight of hens fed the high zinc diet for 6 days and 12 days decreased as compared with body weights prior to being fed the high zinc diet, and body weights increased to day 35 or 23 days after cessation of feeding the high zinc diet (Figure 1B). Similarly, the weights and lengths of oviduct decreased progressively during the induced molting period (days 0 to 12) when egg laying ceased, and rapid growth of the oviduct occurred between days 12 and 35 of this study (Figure 1C and 1D). In the same manner, weights of the ovaries decreased significantly at day 12 as compared with hens on a control diet, and gradually increased during the recrudescence periods to that of normal ovaries (Figure 1E).

### Endocrinological and Histological Changes during Induced Molting and Oviduct Recrudescence

**Figure 2:**
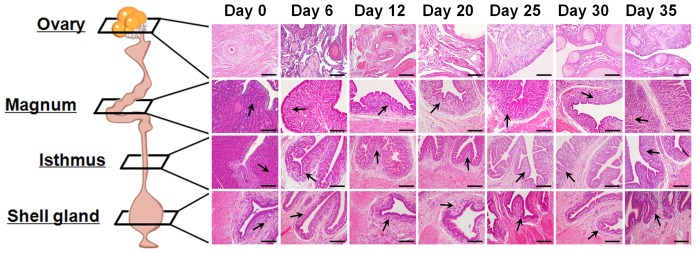
Histological evaluation of ovarian and oviductal tissues at different days of the molting and recrudescence periods. The tissues from the ovary, magnum, isthmus and shell gland were observed following staining with hematoxylin and eosin (H&E). Images were captured at 20× magnification. Arrow: Examples of tubular glands; *Scale bar* represents 100 µm.

We hypothesized that the morphological and functional changes during the molting period were closely related to alterations in the endocrine system. As we expected, concentrations of testosterone, progesterone, estradiol and corticosterone in serum decreased during the induced molting period (Figure 1F, 1G, 1H and 1I) and then increased during the recrudescence period (days 20 to 35). Histological analysis of the tissues revealed regression and remodeling of the ovaries and oviducts in hens undergoing molting or recrudescence that included: dramatic changes in size, involution and formation of tubular glands; regression and recrudescence of ovarian stroma; degeneration of epithelia and reductions in secretions in glands that were followed by gradual recovery during oviduct recrudescence (Figure 2).

### Apoptotic Cell Deaths during Molting and Recrudescence

**Figure 3:**
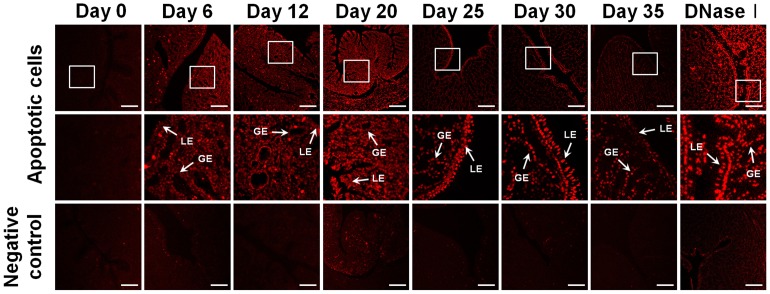
TUNEL (TdT-Mediated dUTP Nick End Labeling) stained cells in the magnum of hens fed a high zinc diet or a normal diet. Endogenous DNA fragmentation in nuclei of cells indicates programmed cell death. For positive controls, tissues were incubated with DNase I prior to labeling procedures. For negative controls, tissues were incubated only in the label solution instead of TUNEL reaction mixture. Images were captured at 20× (top) and 40× (middle) magnification. Legend: LE, luminal epithelium; GE, glandular epithelium; *Scale bar* represents 50 µm.

To detect apoptotic cell death at the single cell level in oviducts of molting or recrudescing hens, we detected changes in DNA fragmentation generated by DNase activity in nuclei during apoptosis (Figure 3). DNA fragments were detected by labeling free 3′-OH termini. We found that most cells in the magnum were TUNEL (TdT-Mediated dUTP Nick End Labeling)-positive starting on day 6 and this condition persisted until day 20. With return to a normal diet, the intensive staining gradually decreased during recrudescence of the oviduct (days 25 to 35).

### Immunohistochemical Staining for Cytokeratin, Vimentin and Proliferating Cell Nuclear Antigen (PCNA)

**Figure 4:**
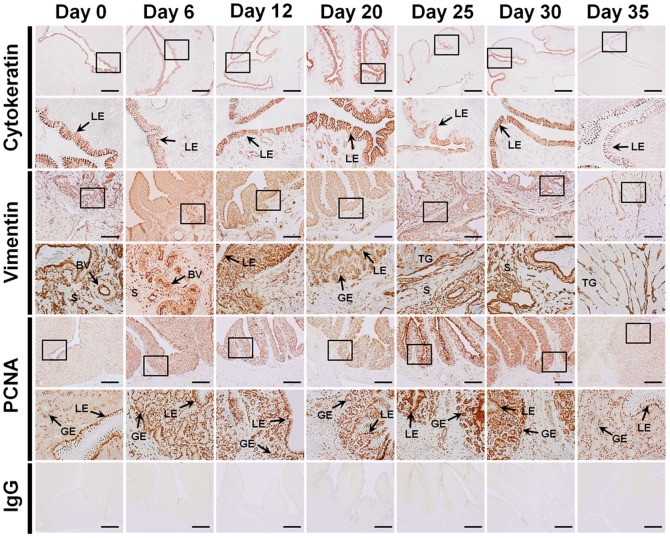
Immunohistochemical staining for detection of cytokeratin, vimentin and PCNA in cells of the magnum from each of the days during the molting and recrudescence periods. For negative controls, tissues were incubated with a nonspecific IgG. Legend: LE, luminal epithelium; GE, glandular epithelium; TG, tubular gland; S; stroma; *Scale bar* represents 100 µm.

Regression and recrudescence of the oviduct was assessed by immunohistochemical analysis for markers of EMT (epithelial-to-mesenchymal transition) and proliferation, that is, cytokeratin (epithelial cell marker), vimentin (mesenchymal cell marker) and PCNA (proliferating cell marker) (Figure 4). Luminal epithelial cells and glandular epithelial cells had abundant amounts of cytokeratin on day 20. Vimentin expression was detected in the endometrial stroma and blood vessels. Extensive expression of vimentin, a mesenchymal cytoskeletal protein, was detected in cells of mesenchymal origin such as endothelial cells and arteries. On days 25 and 30, the relative frequency of PCNA-positive cells increased as compared with other days, and stained cells were detected in the basal region of the luminal and glandular epithelia and stromal cells. By 23 days after cessation of zinc feeding (day 35), the frequency of PCNA-positive cells decreased to that determined for the reproductive tract of laying hens prior to molting (day 0).

### Altered Gene Expression Patterns during Induced Molting and Oviduct Recrudescence

**Figure 5:**
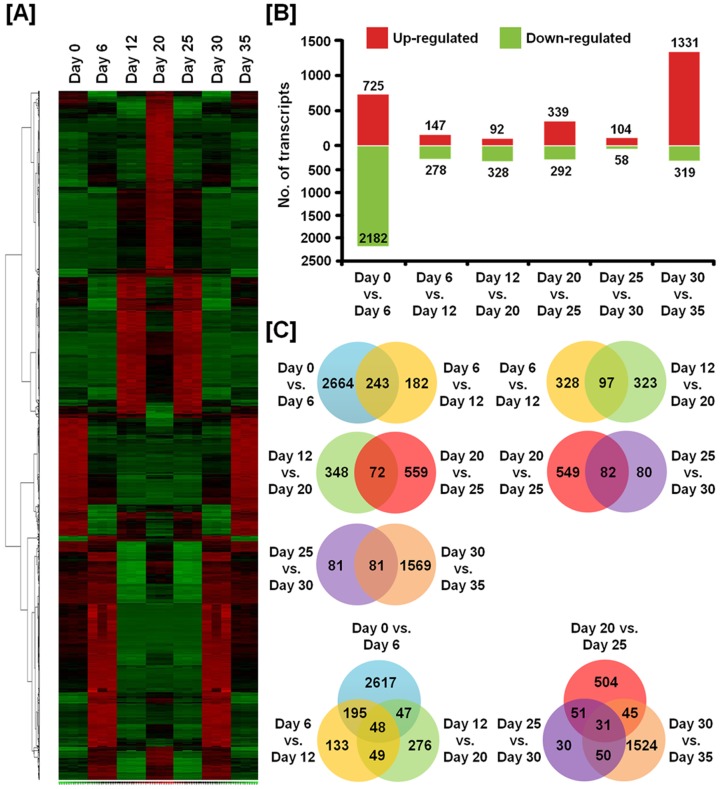
Identification of significant up- or down-regulated genes on each day during regression and regeneration of the oviduct. (A) Heat map of hierarchically clustered genes detected alterations in their expression across the time course of this study. (B) The number of transcripts for which expression increased or decreased significantly in the magnum on different days. Differentially expressed genes were selected using two-sample comparison according to the following criteria: lower bound of 90% confidence interval of fold-change greater than 2.0 and an absolute value of difference between groups means greater than 100. (C) The numbers displayed within the intersections of the circles indicate the number of common genes in the two or three comparisons.

Using affymetrix Genechip microarrays, we compared genome-wide gene expression patterns in the magnum of the oviduct between individual days (day 0 vs. day 6, day 6 vs. day 12, day 12 vs. day 20, day 20 vs. day 25, day 25 vs. day 30, and day 30 vs. day 35) (Figure 5). As illustrated in Figure 5A, clustering analysis of significant genes identified associations of these changes in expression to sets of genes with similar profiles. We used a two-fold change as the experimental cut-off value that would imply significance based on Student's t-test p-values (*p*<0.05). Of the all genes, 725 transcripts were up-regulated and 2182 transcripts were down-regulated more than two-fold at day 0 (normal feeding hens) as compared with day 6 (high zinc feeding hens for 6 days), 147 transcripts were up-regulated and 278 transcripts were down-regulated over two-fold at day 6 as compared with day 12 (high zinc feeding hens for 12 days), and 92 transcripts were up-regulated and 328 transcripts were down-regulated over two-fold at day 12 as compared with day 20 (normal feed for hens for 8 days after cessation of egg production). Furthermore, 339 transcripts were up-regulated and 292 transcripts were down-regulated at day 20 as compared with day 25 (normal feed for hens for 13 days after cessation of egg production), 104 transcripts were up-regulated and 58 transcripts were down-regulated at day 25 as compared with day 30 (normal feed for hens or 18 days after cessation of egg production), and 1331 transcripts were up-regulated and 319 transcripts were down-regulated at day 30 as compared with day 35 (normal feed for hens for 23 days after cessation of egg production) (Figure 5B). The continuously co-regulated genes are presented as Venn diagrams (Figure 5C).

### Functional Categorization of Significantly Altered Genes during Induced Molting and Recrudescence of the Oviduct

**Figure 6:**
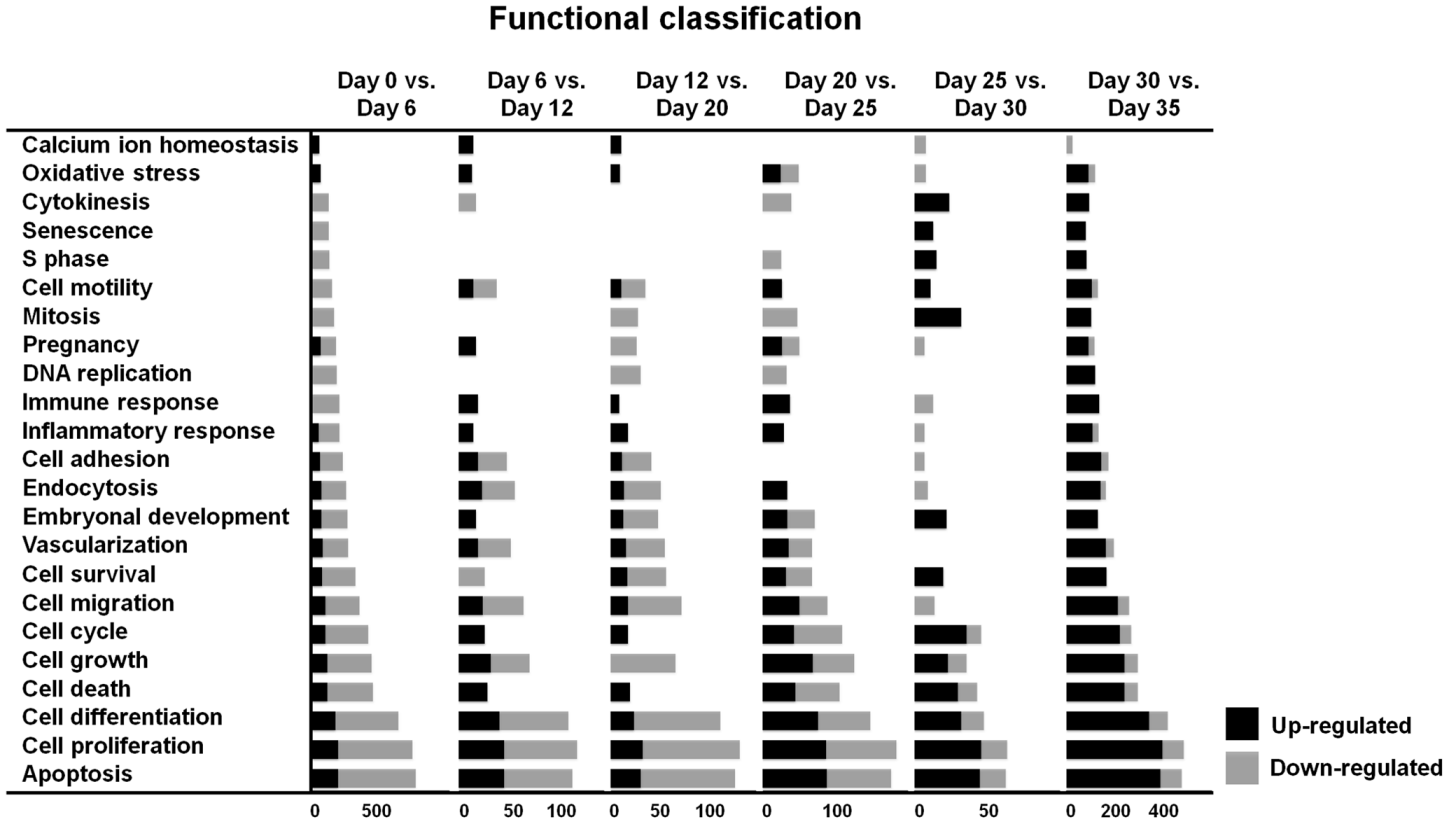
Functional categorization of differentially expressed genes found to be associated with cellular and molecular functions. Genes that changed significantly were assigned to various functional annotations related to cellular processes based on Gene ontology annotation.

We performed functional analysis of genes that were differentially expressed during molting and the recrudescence processes based on their involvement in specific biological processes using the Gene Ontology annotations (Figure 6 and Tables S1-S6). From day 0 through day 35, differentially expressed genes were enriched with functional annotations relating to cellular processes such as apoptosis, cell proliferation and cell differentiation, and most categories were uniformly induced or repressed in their gene expression patterns. In the magnum, between days 0 and 6, days 6 and 12, and days 12 and 20, most genes relating to apoptosis, cell proliferation and cell differentiation were down-regulated. Otherwise, in the magnum between days 20 and 25, days 25 and 30, and days 30 and day 35, most genes relating to apoptosis, cell proliferation and cell differentiation were up-regulated.

### Validation of Selected Genes

**Figure 7:**
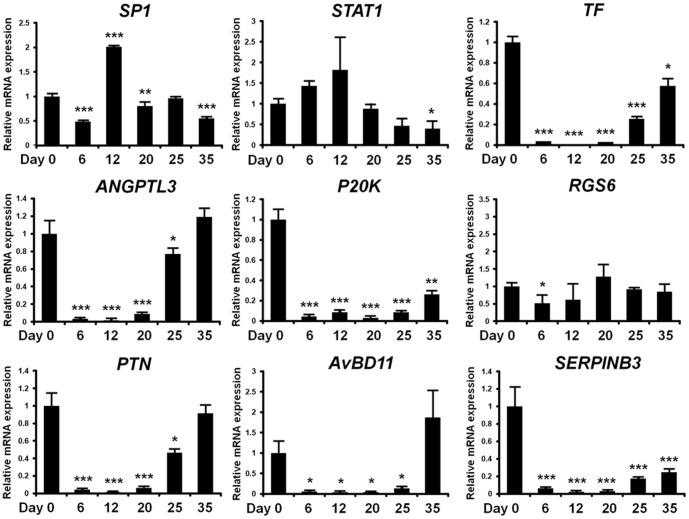
Validation of microarray gene expression data by quantitative RT-PCR analysis. Based on the microarray data, nine genes were selected and their expression pattern confirmed for each pooled RNA sample. All values were normalized to *GAPDH* as the housekeeping gene. Each bar is mean±SEM of three independent experiments. The asterisks denote effects that were significant (***p<0.001, ** *p<*0.01, or * *p<*0.05).

**Figure 8:**
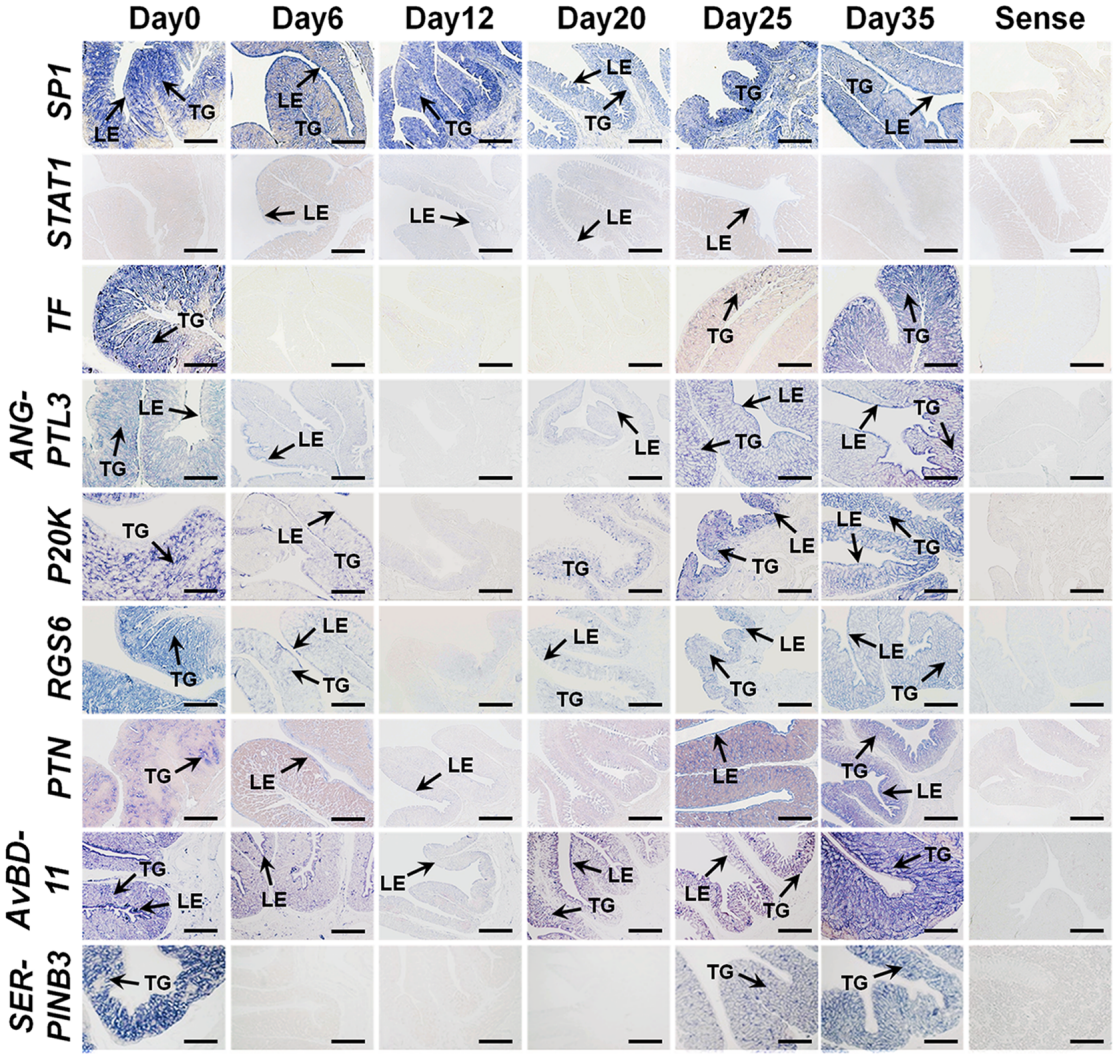
Cell-specific localization of mRNAs for selected genes for which expression changed significantly in the magnum of hens at different days during the pre- and post-molting periods. Expression of *TF*, *PTN*, *ANGPTL3*, *p20K*, *RGS6*, *GAL11* and S*ERPINB3* mRNAs were very abundant in GE and LE of the magnum on Day 0 (normal diet), but expression of those mRNAs were very low at Day 12 (cessation of egg laying and regression of the oviduct). Then, from Days 12 to 35 (normal diet and regeneration of the oviduct), those mRNAs increased gradually in the GE and LE. Legend: TG, tubular gland; LE, luminal epithelium. *Scale bar* represents 100 µm.

Changes in expression of mRNAs for nine selected genes in the magnum from hens fed a control diet (day 0), zinc fed hens (days 6 and 12) and recrudescence hens (days 20, 25, 30 and 35) were analyzed by quantitative RT-PCR analysis. As shown in figure 7, *Sp1* mRNA levels increased 2-fold (*p*<0.001) on day 12 as compared to day 0 and then decreased from day 12 to day 35 (during recrudescence period). *STAT1* mRNA levels increased 1.8-fold (not significant) on day 12 as compare to day 0, and decreased to less than 35% of that on day 0 (*p*<0.05) on day 35. In contrast, expression of *TF*, *ANGPTL3*, *p20K*, *PTN*, *GAL11* and *SERPINB3* mRNAs decreased to less than 0.008- (*p*<0.001), 0.02- (*p*<0.001), 0.08- (*p*<0.001), 0.01- (*p*<0.001), 0.04- (*p*<0.05) and 0.02% (*p*<0.001) of day 0 levels as compared to values on day 12, and from days 12 to 35 (recrudescence period) expression of mRNAs for these genes increased. These mRNA expression patterns were consistent with results from the microarray analysis. The *RGS6* mRNA decreased less than 50% (*p*<0.05) of that on day 0 at day 6, but was not different (*p*>0.05) between days 0 and any other day in the magnum. The cell specific localization of these validated mRNAs was assessed in the magnum of hens at different days during induced molting and recrudescence by *in situ* hybridization analysis (Figure 8). As expected, *TF*, *ANGPTL3*, *p20K*, *RGS6*, *PTN*, *GAL11* and *SERPINB3* mRNAs were detected predominantly in glandular (GE) and luminal (LE) epithelia of the magnum on day 0, but expression of all of these mRNAs decreased to basal levels by day 12 before increasing from days 12 to 35 in GE and LE to that detected on day 0. *STAT1* expression was localized to LE at a very weak basal level. In contrast, *Sp1* expression was generally strong on all experimental days in GE and LE of the magnum.

### Experimental Validation of miRNA-mRNA Target of Selected Genes

**Figure 9:**
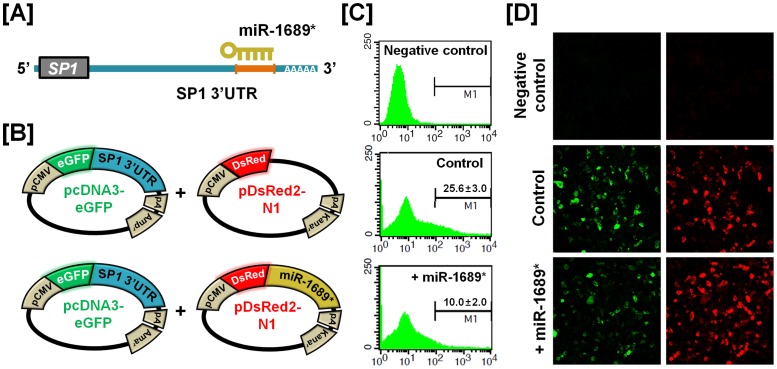
*In vitro* target assay for effects of the chicken miR-1689* on expression of the *Sp1* transcript. (A) Diagram of miR-1689* binding site in the 3′-UTR region of the *Sp1* transcript. (B) The maps of eGFP-Sp1 3′-UTR expressing and DsRed-miR-1689* expressing vector. The Sp1 3′-UTR sequence was subcloned between the eGFP gene and the polyA tail, generating the fusion construct of the *GFP* transcript following the Sp1 3′-UTR (left panel). A miRNA expressing vector was designed to generate *DsRed* and miR-1689* fused transcript or only *DsRed* transcript for the experimental control (right panel). (C and D) After co-transfection of pcDNA-eGFP-Sp1 3′-UTR and pcDNA-DsRed-miR-1689* into 293FT cells, the fluorescence signals of GFP and DsRed were detected using FACS (C) and fluorescent microscopy (D). In the panel C, a gated event value from histogram statistics was used to find statistical percentages of the positive events (GFP-expressing cells) and the numbers indicate mean±SEM of three independent experiments.

**Figure 10:**
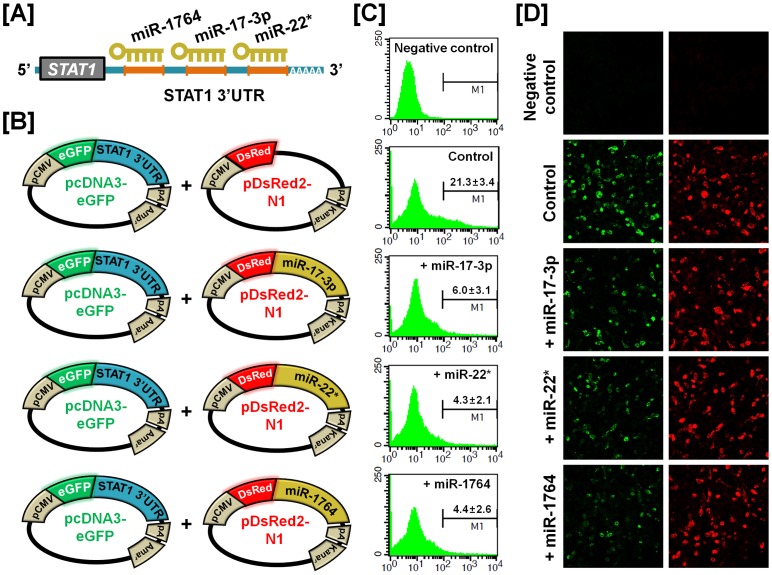
*In vitro* target assay for effects of chicken miR-17-3p, miR-22* and *miR-1764* on expression of the *STAT1* transcript. (A) Diagram of miRNAs binding sites in the 3′-UTR region of the *STAT1* transcript. (B) The maps of eGFP-STAT1 3′-UTR expressing and DsRed-each miRNA expressing vector. The STAT1 3′-UTR sequence was subcloned between the eGFP gene and the polyA tail, generating the fusion construct of the *GFP* transcript following the STAT1 3′-UTR (left panel). A miRNA expressing vector was designed to generate *DsRed* and each miRNA fused transcript or only *DsRed* transcript for the experimental control (right panel). (C and D) After co-transfection of pcDNA-eGFP-STAT1 3′-UTR and pcDNA-DsRed-each miRNA into 293FT cells, the fluorescence signals of GFP and DsRed were detected using FACS (C) and fluorescent microscopy (D). In panel C, a gated event value from histogram statistics was used to find statistical percentages of the positive events (GFP-expressing cells) and the numbers indicate mean±SEM of three independent experiments.

**Figure 11:**
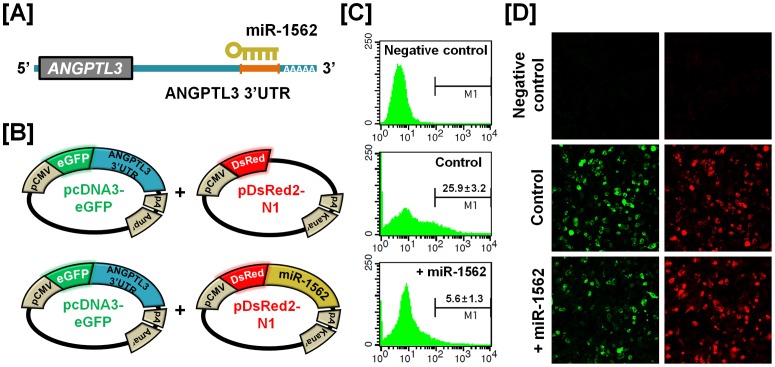
*In vitro* target assay for effects of the chicken *miR-1562* on expression of the *ANGPTL3* transcript. (A) Diagram of the miRNA binding site in the 3′-UTR region of the *ANGPTL3* transcript. (B) The maps of eGFP-ANGPTL3 3′-UTR expressing and DsRed-each miRNA expressing vector. The ANGPTL3 3′-UTR sequence was subcloned between the eGFP gene and the polyA tail, generating the fusion construct of the *GFP* transcript following the ANGPTL3 3′-UTR (left panel). A miRNA expressing vector was designed to generate *DsRed* and *miR-1562* fused transcript or only *DsRed* transcript for the experimental control (right panel). (C and D) After co-transfection of pcDNA-eGFP-ANGPTL3 3′-UTR and pcDNA-DsRed-miR-1562 into 293FT cells, the fluorescence signals of GFP and DsRed were detected using FACS (C) and fluorescent microscopy (D). In panel C, a gated event value from histogram statistics was used to find statistical percentages of the positive events (GFP-expressing cells) and the numbers indicate mean±SEM of three independent experiments.

**Figure 12:**
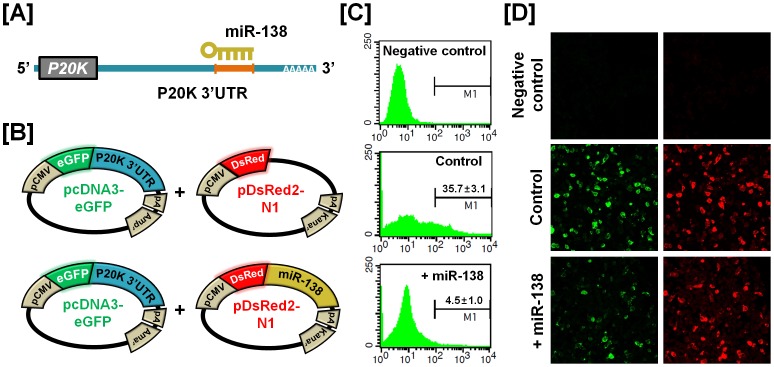
*In vitro* target assay for expression of the chicken *miR-138* on expression of the *p20K* transcript. (A) Diagram of miRNA binding site in the 3′-UTR region of the *p20K* transcript. (B) The maps of eGFP-p20K 3′-UTR expressing and DsRed-miR-138 expressing vector. The p20K 3′-UTR sequence was subcloned between the eGFP gene and the polyA tail, generating the fusion construct of the *GFP* transcript following the p20K 3′-UTR (left panel). A miRNA expressing vector was designed to generate *DsRed* and miR-138 fused transcript or only *DsRed* transcript for the experimental control (right panel). (C and D) After co-transfection of pcDNA-eGFP-p20K 3′-UTR and pcDNA-DsRed-miR-138 into 293FT cells, the fluorescence signals of GFP and DsRed were detected using FACS (C) and fluorescent microscopy (D). In panel C, a gated event value from histogram statistics was used to find statistical percentages of the positive events (GFP-expressing cells) and the numbers indicate mean±SEM of three independent experiments.

To determine the post-transcriptional regulation of selected genes by microRNA (miRNA), we performed miRNA target validation assays. The miRNA target prediction database (miRDB; http://mirdb.org/miRDB/) predicts potential miRNA binding sites on specific mRNA targets (Figure 9A, 10A, 11A and 12A). The 3′UTR of the target gene of interest was cloned in immediately downstream of the green fluorescent protein open reading frame sequence, and miRNAs for each specific mRNA target site was cloned in downstream of the red fluorescent protein open reading frame sequence. The recombinant plasmids were co-transfected into 293FT cells, and for experimental control, just DsRed constructs that do not express the miRNA of interest are transfected into cells (Figure 9B, 10B, 11B and 12B). The percentage of GFP-expressing cells and total GFP intensities of multiple cells were measured using fluorescence microscopy and fluorescence activated cell sorting (FACS). The binding of a predicted miRNA to its specific target site on the 3′UTR reduces production of GFP thereby repressing GFP expression which can be measured and compared to a control. As illustrated in Figures 9C and 9D, miR-1689* reduced the intensity of GFP signal (25.6±3.0% in control vs. 10.0±2.0% in miR-1689*; *p*<0.01) suggesting that chicken miR-1689* interacted with the predicted binding site harbored within the 3′UTR of the *Sp1* gene. In the presence of miR-17-3p, miR-22* and *miR-1764* which target the 3′UTR of *STAT1*, the intensities of GFP signals (21.3±3.4% in control vs. 6.0±3.1% in *miR-17-3p*; *p<*0.01, 4.3±2.1% in miR-22*; *p<*0.01 and 4.4±2.6% in *miR-1764*; *p*<0.01) were decreased, respectively (Figure 10C and 10D). Similarly, *miR-1562* targeting the 3′UTR of *ANGPTL3* 3′UTR and *miR-138* targeting the 3′UTR of *p20K* reduced the intensity of the GFP signal (25.9±3.2% in control vs. 5.6±1.3% in *miR-1562*; *p<*0.001 and 35.7±3.1% in control vs. 4.5±1.0% in *miR-138*; *p*<0.001), suggesting that chicken *miR-1562* and *miR-138* bind within the 3′UTR of *ANGPTL3* and *p20K* genes, respectively (Figure 11C, 11D, 12C and 12D).

## Discussion

The molting process leads to dynamic changes in morphology, physiology and function of the reproductive tract of laying hens. In the present study, results from differential gene profiling identified global genes that potentially regulate oviductal regression and recrudescence during and following molting. Our results also revealed spatial and temporal expression of selected candidate genes in the magnum during tissue remodeling and post-transcriptional regulation of these genes by specific chicken miRNAs. These results support our hypothesis that complete recovery of the reproductive system following molting involves complex tissue remodeling that is reversible and involves specific changes in gene expression and molecular aspects.

In order to identify candidate genes that potentially regulate oviduct regression or tissue remodeling, we established the chicken *in vivo* model for induced molting and post-molting recrudescence (Figure 1). For induction of molting, we fed hens a diet enriched in zinc as previously reported [13], [22], [23], which led to complete cessation of egg production within 12 days. After the complete cessation of egg production (day 12), molted hens were removed from the high zinc diets and they resumed egg production within 23 days, which is similar to results reported by Scott *et al*. [23], [24].

Along with changes in egg production, dramatic regression of oviducts and ovaries occurred in response to the high zinc diet, but this was followed by recrudescence after the hens were returned to a normal diet. Weights of the whole body and reproductive organs, as well as length of the oviduct were measured, all of which showed similar V-shaped patterns. The change in overall body weight is directly associated with in weights of liver, muscle, adipose tissue, and involution of reproductive tissues [25], [26]. The extent of body loss during molting is a key factor for successful post-molting improvements in egg quality and egg production [27]. The decreases and gains in ovarian and oviductal weights may be related to the overall rejuvenation of the hen associated with increases in metabolic processes of many tissues [14], [28]. The overall regeneration processes included recovery of atretic follicles, release of egg yolk materials and ovarian steroids, and increases in weights of reproductive tissues, as well as cellular and tissue morphology [2].

Previous studies have focused mainly on the period of regression of the oviduct, so the physiological mechanisms that regulate recrudescence processes are not understood to date. In the current study, however, changes in circulating concentrations of testosterone, progesterone, estradiol and corticosterone were determined in hens undergoing recrudescence (Figure 1F, 1G, 1H and 1I). Hoshino *et al* reported that concentrations of progesterone and estradiol decrease during molting due to low activities of enzymes for ovarian steroidogenesis [29]. The decrease in selected ovarian steroids leads to apoptosis in the oviduct and regression of the ovary during molting in hens [30]. During the recrudescence period, luteinizing hormone and follicle stimulating hormone levels increase, which stimulates ovarian development and their secretion of estradiol and progesterone to stimulate growth of the oviduct. Circulating levels of testosterone and corticosterone are transitorily greater in hens during molting than in normal laying hens, and they decline as the molting progresses and increases with resumption of egg laying [2], [29]. In the present study, corticosterone levels increased upon return to the normal diet, which is considered to be associated with changes in activity of the hypothalamic-pituitary-adrenal (HPA) axis following replenishment of energy reserves in the body [31]. And, although the function of testosterone in the recrudescence process is obscure, the increase in testosterone may influence oviductal regeneration, as well as thermoregulation.

Histomorphological changes in the magnum size, tubular glands and ovarian stroma during tissue regression and regeneration were dramatic (Figure 2). Regression of the reproductive organs during induced molting is achieved through apoptotic processes [32]. We observed that induction of molting by feeding a high zinc diet caused apoptotic cell death in all cell types of the magnum (Figure 3). Zinc is an endogenous regulator of apoptosis via involvement in several complex processes such as the alterations in caspase activity, and regulation of cytokine expression and hormone levels [13], [33]. We also observed cytokeratin-, vimentin-, and PCNA-positive cells in the magnum of hens undergoing oviductal regression and recrudescence (Figure 4). Epithelial-to-mesenchymal transition (EMT) endows cells with migratory and invasive properties, and plays an important role in developmental processes including tissue repair and differentiation [34]. Previously, Heryanto *et al* suggested that the tubular glands were generated by the invasion and cytodifferentiation of the mucosal epithelium and old glandular cells were replaced with newly derived GE during oviductal tissue remodeling [35], [36]. Our results suggest that up-regulation of cytokeratin is a defining factor for initiation of regeneration and plays a significant role in initial tissue remodeling of the reproductive tract. And the relative high frequency of PCNA-positive cells in the developing magnum between days 25 and 30 suggests active proliferation during the gradual recrudescence of reproductive organs. We assume that proliferation of oviductal cells between days 25 and 30 is likely induced by ovarian steroids following resumption of ovarian steroidogenesis. These morphological and physiological findings of the present study indicate that the laying hen undergoing molting and recovery from molting is a highly efficient animal model for research on regression and recrudescence of the mammalian reproductive system.

Using cDNA microarray analysis of tissues from this *in vivo* model, we identified a large number of genes that are differentially regulated in the magnum portion which undergoes the most dramatic changes during reproductive tissue remodeling (Figure 5). Interestingly, we observed expression of about 20 genes including *transferrin (TF)*, *angiopoietin-like 3 (ANGPTL3)*, *p20K*, *regulator of G-protein signaling 6 (RGS6)*, *pleiotrophin (PTN)*, *avian beta-defensin11 (AvBD11)* and *SERPINB3* that decreased gradually during molting and increased following regeneration of the oviduct. And, through pathway analysis, these genes are considered to be commonly regulated by *Sp1* and *STAT1*. The expression levels of mRNAs for nine interrelated genes during oviductal remodeling were analyzed to validate results from the cDNA microarray analysis. Sp1 is a ubiquitous transcription factor expressed in diverse cell types and believed to bind to GC/GT-boxes on promoters and other regulatory sequences of genes [37], [38]. It has diverse functions via synergistically activating or repressing transcription of genes involved in various biological processes [39]–[50]. STAT1, similar to Sp1, is a latent ubiquitous transcription factor that regulates expression of a wide range of downstream genes in response to cytokines and growth factors [51], [52]. STAT1 translocate to the nucleus and bind to conserved enhancer elements that lead to transcriptional activation of immediate early response genes [53]. Although *Sp1* and *STAT1* are expressed ubiquitously, the expression of those genes changes according to developmental stages or cell type implying specific roles in developmental processes [54]. The important phenotypic changes during oviductal regression and recrudescence would appear to result from a finely balanced transcriptional response by several transcription factors including Sp1 and STAT1.

The third gene of interest, *TF*, encodes for an iron-binding protein that absorbs dietary iron from the gut and transports it throughout the body [55]. In chickens, this gene is synthesized by both liver cells and oviduct cells, and stimulated by estrogen or dietary iron deficiency [56]-[58]. The expression of this gene increased during oviduct regeneration indicating that it might be affect changes in gross metabolic rates. It is well known that iron is essential for various metabolic processes including DNA synthesis, oxygen transport and the electron transport system in a wide range of tissues. Dramatic tissue remodeling involving degeneration and regeneration of female reproductive organs is necessarily coupled with angiogenic processes. ANGPTL3 has features common to angiopoietins that play important roles during angiogenesis as vascular endothelial cell-specific growth factors [59]–[61]. In the present study, there was a remarkable increase of *ANGPTL3* mRNA during oviductal recrudescence which implies a requirement for re-growth of the vascular network in developing tissue. The fifth gene of interest, p20K, encodes for a small protein cloned from chicken embryo fibroblasts, and its transcription is induced in the growth arrested phase of the cell cycle such as serum-deprivation and contact-inhibition [62]. RGS6, a member of regulator of G protein signaling (RGS) proteins, negatively regulates G protein signaling by activating the GTPase activity [63], [64]. Although the precise physiological roles of p20K and RGS6 are unknown, they are linked to cellular stress responses and possess abilities to induce cell cycle arrest and apoptosis [65]–[67].

Results from our previous studies indicate that *PTN*, *AvBD11* and *SERPINB3* are estrogen-stimulated genes during development of the chicken oviduct [68]-[70]. PTN, also known as heparin binding growth factor 8 (HBGF-8), is a developmentally regulated growth factor which is widely distributed in various tissues [71], [72]. In chickens, it especially plays important roles in differentiation and morphogenesis of the embryo by promoting cell growth and migration [72]–[75]. AvBD11, a component of egg-white proteins, is an antimicrobial peptide that plays a role in innate immunity [76]–[78]. We reported that AvBD11 may influence oviduct development and protect the chicken embryo from pathogenic microorganisms [19], [79]. SERPINB3 was first identified in squamous cell carcinoma of the human uterus and, in chickens, it plays a crucial role in formation of egg yolk and egg white [80], [81]. Complicated actions of estrogen in various biological processes affect physiological changes through diverse regulatory cascades by trans-activating transcription factors and inducing expression of various target genes. Because estrogen produced by the ovary may orchestrate the expression of *PTN*, *AvBD11* and *SERPINB3*, temporal patterns of expression of these genes during molting and oviduct recrudescence may be closely related to changes in estrogen levels following withdrawal and restart of ovarian steroidogenesis. Taken together, tissue-specific and developmentally regulated expression of these nine genes indicates that they have regulatory functions during reproductive tissue remodeling and development of the oviduct in chickens.

For dramatic phenotypic changes in the reproductive tract of female chickens (see Figure 1), microRNA (miRNA)-mediated posttranscriptional regulation of genes can effect changes in expression of related genes at the cellular level [82]. To further investigate the possible involvement of miRNA-mediated posttranscriptional regulation during regeneration of the chicken oviduct, we performed screening to identify potential miRNAs targeting *Sp1*, *STAT1*, *ANGPTL3* and *p20K* transcripts. As shown in Figures 9–12, specific chicken miRNAs reduced expression of *Sp1*, *STAT1*, *ANGPTL3* and *p20K*, respectively. Similarly, we previously demonstrated inhibitory effects of chicken miRNAs on *PTN*, *AvBD11* and *SERPINB3* gene expression [68], [69], [83]. These results indicate that specific chicken miRNAs interact with transcripts for at least these seven genes that determine oviduct development and remodeling, and regulate their expression post-transcriptionally during remodeling of reproductive organs. Emerging evidence from animal models suggests that microRNAs are expressed within the female reproductive tract and that post-transcriptional regulation of genes through miRNAs is essential to regulate cellular pathways for proper development and function of the organs. Even though miRNA-mediated gene regulation plays an important role in the synthesis of all proteins required for the rapid phenotypic changes occurring in the female reproductive tract, much less is known about it in oviductal tissue regression or recrudescence. Also, the identity and functional analysis of individual miRNAs expressed in the reproductive tract of chickens, and the identification of their specific mRNA targets are just now being identified. Although the significance of this regulation by the chicken miRNAs remains to be determined, results of the present study will be helpful in elucidating regulation of mechanisms whereby the suggested miRNAs participate in oviductal regression and remodeling processes. These results will also provide new research approaches and insights into how post-transcriptional regulation by miRNAs enhances reproductive efficiency and/or development of the reproductive organs.

In summary, global gene expression profiles from our present microarray study using a well-established *in vivo* model for molting and regeneration of chicken oviductal tissue identified new molecular candidates regulating this process. Our findings also revealed the biological significance of genetic and miRNA-mediated epigenetic regulation in morphological- and functional recrudescence of the reproductive tract in chickens. These findings provide new clues for further studies to determine regulatory roles of novel developmentally related genes and molecular mechanism for reproductive tissue remodeling in chickens.

## Materials and Methods

### Experimental Animals and Animal Care

The experimental use of chickens for this study was approved by the Animal Care and Use Committee of Dankook University. White Leghorn (WL) chickens were exposed to a light regimen of 15 h light and 9 h dark with *ad libitum* access to feed and water, and subjected to standard poultry husbandry guidelines.

### Molting and Recrudescence Induction

Molting of laying hens was induced as described previously that involves adding 20,000 ppm zinc to the diet to effectively reduce feed-intake and induces molting [26], [84]. Briefly, molting was induced by feeding hens in the zinc-fed group a diet containing high zinc (mixed 252 g zinc oxide per 10 kg feed to achieve a final concentration of 20,000 ppm of zinc). Laying hens in the molting group completely ceased egg production within 12 days after feeding the high zinc-diet. The 35 laying hens (47-week-old) were divided into two larger groups, including molting-progressing or post-molting-progressing group, and kept in individual cages. The molting group was divided into three subgroups based on the number of days of feeding the high zinc diet (normal feeding group, 6 days and 12 days after onset of zinc feeding). The recrudescence (post-molting) group was divided into four subgroups based on the number of normal feeding days after complete cessation of egg laying and initiation of feeding a normal commercial diet: 20, 25, 30 or 35 days after onset of zinc feeding or 8, 13, 18 or 23 normal feeding days after cessation of egg production and removal from the high zinc diet.

### Tissue and Blood Sample Collection

Hens (n = 5 per time point) in each subgroup (0, 6, 12, 20, 25, 30 and 35 days after onset of zinc feeding) were bled and serum was obtained and stored at −80°C until further use. After euthanizing the hens using 60%–70% carbon dioxide, the ovary and oviduct were removed and measured for length and weight at each assigned day. Oviduct weight was taken after removing the egg, if present, from the oviduct. Portions of the ovary, magnum, isthmus and shell gland of each oviduct from each hen at each time point were cut into 10- to 15-mm pieces and either: 1) frozen immediately in liquid nitrogen and stored at −80°C until analyzed; or 2) fixed in freshly prepared 4% paraformaldehyde in PBS (pH 7.4). After 24 h, tissues fixed in 4% paraformaldehyde were changed to 70% ethanol for 24 h and then dehydrated and embedded in Paraplast-Plus (Leica Microsystems, Wetzlar, Germany). Paraffin-embedded tissues were sectioned at 5 µm and stained with hematoxylin and eosin. Histomorphological changes of the stained tissue sections were evaluated using a microscope (Leica Microsystems, DM3000, Germany).

### Estimation of Serum Hormones

Concentrations of testosterone, progesterone, estradiol and corticosterone in serum from individual hens during molting and the recrudescence processes were determined by EIA kits for testosterone (catalog number EA78; Oxford Biomedical Research, MI, USA), progesterone (catalog number EA74; Oxford Biomedical Research, MI, USA), estradiol (catalog number EA70; Oxford Biomedical Research, MI, USA) and corticosterone (catalog number EA66; Oxford Biomedical Research, MI, USA). Each sample was assayed in duplicate according to the manufacturer's directions. Briefly, arterial blood samples from each hen were centrifuged at 3000 g for 30 min at 4°C and plasma separated and incubated with diluted hormone conjugate for 1 h. Unbounded hormone was removed through a washing process and bound hormone conjugate was detected following the addition of substrate which generates an optimal color. Quantitative results were obtained by measuring and comparing the absorbance reading of the samples against the standards with a microplate reader (Bio-Rad, CA, USA) at 450 nm (for samples) or 650 nm (for standards), respectively.

### RNA Isolation

Total RNA was isolated from frozen magnum tissues from each time point using Trizol reagent (Invitrogen, Carlsbad, CA) according to manufacturer's recommendations. The quantity and quality of total RNA was determined by spectrometry and denaturing agarose gel electrophoresis, respectively.

### Microarray Analysis

Microarray analysis was performed using Affymetrix GeneChip® Chicken Genome Arrays (Affymetrix, Santa Clara, CA, USA) which contained 30,000 probes corresponding to known chicken genes. For microarray analysis, total RNAs were extracted from the magnum from each of the four hens at each time point (0, 6, 12, 20, 25, 30 and 35 days after onset of zinc feeding) and purified using an RNeasy Mini Kit (Qiagen, Valencia, CA, USA). Data were generated by InsilicoGen (Suwon, Korea) and dChip software was used for the analysis. All experiments were performed using four independent RNA pools from each of the four hens at each time point and three independent microarray chips. The signal intensity of each spot was calculated, and then differentially expressed genes were identified by the net intensity ratios between the two test groups. The student t-test p-values were used to compare intensities between the two groups. A p-value of 0.05 was used as threshold value for statistical significance. Next, pathway analysis was performed using Pathway Studio software to classify specific signaling pathways and gene regulation maps. Gene Ontology (GO) analysis was conducted to further clarify the functional roles of differentially expressed genes and classify them based on their functions in cellular processes using GeneSpring software (Silicon Genetics, Agilent Technologies, Palo Alto, CA).

### Quantitative RT-PCR Analysis

Complementary DNA was synthesized using total RNA extracted from magnum and AccuPower® RT PreMix (Bioneer, Daejeon, Korea). Gene expression levels were measured using SYBR® Green (Sigma, St. Louis, MO, USA) and a StepOnePlus™ Real-Time PCR System (Applied Biosystems, Foster City, CA, USA). The *GAPDH* gene was simultaneously analyzed as a control and used for normalization for variation in loading. Each target gene and *GAPDH* was analyzed in triplicate. Using the standard curve method, we determined the level of expression of the examined genes using the standard curves and C_T_ values, and normalized them based on *GAPDH* expression. The PCR conditions were 95°C for 3 min, followed by 40 cycles at 95°C for 30 sec, 60°C for 30 sec, and 72°C for 30 sec using a melting curve program (increasing the temperature from 55°C to 95°C at a rate of 0.5°C per 10 sec) and continuous fluorescence measurement. ROX dye (Invitrogen) was used as a negative control for the fluorescence measurements. Sequence-specific products were identified by generating a melting curve in which the C_T_ value represented the cycle number at which a fluorescent signal was statistically greater than background, and relative gene expression was quantified using the 2^–ΔΔCT^ method [85]. For the control, the relative quantification of gene expression was normalized to the C_T_ of the Day 0 magnum.

### Detection of Apoptotic Cell Death

Embedded and sectioned tissues were deparaffinized in xylene and washed in 0.1 M PBS. For immunostaining, sections were then permeabilized in proteinase K, and subjected to TUNEL (terminal deoxynucleotidyl transferase–mediated dUTP-fluoroscein nick-end labeling) staining mixture using the In Situ Cell Death Detection kit, TMR red (Roche Diagnostics, Canada) for 1 h at 37°C. As negative controls, tissues were incubated only in the labeling solution instead of the TUNEL reaction mixture. For positive controls, tissues were treated with DNaseI for 10 min at room temperature to induce DNA strand breaks before TUNEL initiating labeling procedures. Fluorescence was detected using a confocal microscope LSM710 (Carl Zeiss) fitted with a digital microscope camera AxioCam using Zen 2009 software.

### *In Situ* Hybridization Analysis

For hybridization probes, PCR products were generated from cDNA with the primers used for RT-PCR analysis. The products were gel-extracted and cloned into TOPO® vector (Invitrogen). After verification of the sequences, plasmids containing the correct gene sequences were amplified with T7- and SP6-specific primers (T7:5′-TGT AAT ACG ACT CAC TAT AGG G-3′; SP6:5′-CTA TTT AGG TGA CAC TAT AGA AT-3′) then digoxigenin (DIG)-labeled RNA probes were transcribed using a DIG RNA labeling kit (Roche Applied Science, Indianapolis, IN). Tissues were collected and fixed in 4% paraformaldehyde, embedded in paraffin and sectioned at 5 µm on APES-treated (silanized) slides. The sections were then deparaffinized in xylene and rehydrated to diethylpyrocarbonate (DEPC)-treated water through a graded series of alcohol. The sections were treated with 1% Triton X-100 in PBS for 20 min and washed two times in DEPC-treated PBS. After washing in DEPC-treated PBS, they were digested with 5 µg/ml proteinase K (Sigma) in TE buffer (100 mM Tris-HCl, 50 mM EDTA, pH 8.0) at 37°C. Paraffin-embedded tissue sections were incubated twice for 5 min each in DEPC-treated PBS and incubated in TEA buffer (0.1 M triethanolamine) containing 0.25% (v/v) acetic anhydride. The sections were next incubated in a prehybridization mixture containing 50% formamide and 4× standard saline citrate (SSC) for at least 10 min at room temperature. After prehybridization, the sections were incubated with a hybridization mixture containing 40% formamide, 4× SSC, 10% dextran sulfate sodium salt, 10 mM DTT, 1 mg/ml yeast tRNA, 1 mg/ml salmon sperm DNA, 0.02% Ficoll, 0.02% polyvinylpyrrolidone, 0.2 mg/ml RNase-free bovine serum albumin and denatured DIG-labeled cRNA probe overnight at 42°C in a humidified chamber. After hybridization, sections were washed for 15 min in 2× SSC at 37°C, 15 min in 1× SSC at 37°C, 30 min in NTE buffer (10 mM Tris, 500 mM NaCl and 1 mM EDTA) at 37°C and 30 min in 0.1× SSC at 37°C. After blocking with a 2% normal sheep serum (Santa Cruz Biotechnology, INC.), the sections were incubated overnight with sheep anti-DIG antibody conjugated to alkaline phosphatase (Roche). The signal was visualized by exposure to a solution containing 0.4 mM 5-bromo-4-chloro-3-indolyl phosphate, 0.4 mM nitroblue tetrazolium, and 2 mM levamisole (Sigma).

### Immunohistochemistry

Immunohistochemical localization of cytokeratin, vimentin and PCNA protein in pre-molting and post-molting magnum from hens was performed as described previously [86] using an anti-mouse cytokeratin monoclonal antibody (catalog number MAB3412; Millipore, MA, USA) at a final dilution of 1∶500 (2 µg/ml), anti-mouse vimentin monoclonal antibody (catalog number CBL202; Millipore, MA, USA) at a final dilution of 1∶100 (0.5 µg/ml) and an anti-mouse PCNA monoclonal antibody (catalog number sc-56; Santa Cruz Biotechnology, CA, USA) at a final dilution of 1∶1000 (1 µg/ml). Antigen retrieval was performed using the boiling citrate method as described previously [86]. Negative controls included substitution of the primary antibody with purified non-immune mouse IgG at the same final concentration.

### MicroRNA Target Validation Assay

The 3′-UTRs of *Sp1*, *STAT1*, *ANGPTL3* and *p20K* were cloned and confirmed by sequencing. Each 3′-UTR was subcloned between the eGFP gene and the bovine growth hormone poly-A tail in pcDNA3eGFP (Clontech, Mountain View, CA) to generate the eGFP-miRNA target 3′-UTR (pcDNA-eGFP-3′UTR) fusion constructs. For the dual fluorescence reporter assay, the fusion contained the *DsRed* gene and either miR-1689* for *Sp1*; miR-17-3p, miR-22* or *miR-1764* for *STAT1*; *miR-1562* for *ANGPTL3* and *miR-138* for *p20K* which were designed to be co-expressed under control of the CMV promoter (pcDNA-DsRed-miRNA). The pcDNA-eGFP-3′UTR and pcDNA-DsRed-miRNA (4 µg) were co-transfected into 293FT cells using the calcium phosphate method. When the DsRed-miRNA is expressed and binds to the target site of the 3′-UTR downstream of the *GFP* transcript, green fluorescence intensity decreases due to degradation of the *GFP* transcript. At 48 h post-transfection, dual fluorescence was detected by fluorescence microscopy and calculated by FACSCalibur flow cytometry (BD Biosciences). For flow cytometry, the cells were fixed in 4% paraformaldehyde and analyzed using FlowJo software (Tree Star Inc., Ashland, OR).

### Statistical Analyses

Data for quantitative PCR were subjected to analysis of variance (ANOVA) according to the general linear model (PROC-GLM) of the SAS program (SAS Institute, Cary, NC) to determine whether differential gene expression during the molting and recrudescence periods were significant. Data are presented as mean±SEM unless otherwise stated.

## Supporting Information

Table S1 Functional categorization of genes changed in the magnum between day 0 and day 6 during the molting period.
(PDF)

Table S2 Functional categorization of genes changed in the magnum between day 6 and day 12 during the molting period.
(PDF)

Table S3 Functional categorization of genes changed in the magnum between day 12 and day 20 during the molting period.
(PDF)

Table S4 Functional categorization of genes changed in the magnum between day 20 and day 25 during the molting period.
(PDF)

Table S5 Functional categorization of genes changed in the magnum between day 25 and day 30 during the molting period.
(PDF)

Table S6 Functional categorization of genes changed in the magnum between day 30 and day 35 during the molting period.
(PDF)
