# Supplementary material for: Recrudescence Mechanisms and Gene Expression Profile of the Reproductive Tracts from Chickens during the Molting Period
Source: PLoS One. 2013 Oct 1;8(10):e76784. doi: 10.1371/journal.pone.0076784 (PMC3788108; doi:10.1371/journal.pone.0076784)
Supplement: Table S1 — Functional categorization of genes changed in the magnum between day 0 and day 6 during the molting period. (PDF) [file pone.0076784.s001.pdf]

Table S1. Functional categorization of genes changed in the magnum between day 0 and day 6 during the molting period.

Category	Day 0 vs. Day 6	Molecules	p-value
	up	SLC5A1,F2,SRC,NPY,ATP2B2,KNG1,MAPK8,TFF3,IL6,MME,CA2,SLC4A2,CAPN2,AQP3,SLIT3,ATP2B4,PTGDS,SELP,RB1,MTOR,TF,CAV1,CAMK4,HSP90B1,TACR1,AQP5,HSPA5,DUSP6,COL2A1,BDNF,ALOX5,NTF3,DUSP1,NR4A3,BNIP3,UTS2,INSR,IAPP,STX1A,ADORA1,CA4,APOA4,HYOU1,ID1,RGS4,PPARD,PTGS1,ANK2,P4HB,FSHR,NRG1,PTH1R,HOMER1,ADORA2A,CNR1,CA9,GADD45B,SKP2,NCOA3,APOD,RARRES2,NCS1,CYP7A1,MMP1,TGFB3,XBP1,PRDX6,PGGT1B,GJB1,LTF,ACE,PRLR,ENPP2,S1PR5,MSI1,STK11,GNAI1,ERP44,LYZ,SFTPA1,ADA,ACTG2,MUC1,GCH1,FABP3,SFRP4,EEF1A1,BMPR2,GADD45G,PRKAR2B,STK17A,PRDX5,CTH,HOMER2,PROKR2,RCAN1,ENPEP,OGDH,PDCD1,PRDX4,BECN1,Sprr1a,TEC,RHOB,RRM2B,XPO1,UCHL1,MAOB,PHB,GAB2,GFI1B,MYCN,STEAP3,MKL1,AMD1,STK25,TNFRSF6B,PEMT,PTN,CHIA,MITF,EPHX1,AMH,GREM1,HHAT,FKBP5,SLCO2A1,PLK3,NID1,NOV,PAEP,SNCA,DHCR24,NFIL3,ETV4,HERPUD1,DAG1,SOX7,RPS27L,RPLP0,SEPP1,BHLHA15,EPS8,CREB3L2,ASNS,RDX,SGCG,PELP1,POLH,FERMT2,NRCAM,GPR87,DEFB1,PDIA3,NDC80,RCAN2,SEMA3C,CACNA2D2,ADCY10,NRG2,CRMP1,APLP2,PSME3,GPR37,CNTN2,MTMR9,TPD52,NUTF2,GRM7,PES1,DNAJB9,CREB3,DAB2IP,MRAS,RDH12,SCN3B,RASSF6,MIB2,DNAJB11,ALG3,MFF,SERPINB3,PSPH,NARS,DPYSL4,CA6,ITPK1,NAIF1,CHAC1	9.12708E-08

Apoptosis	down	CEBPB,EGFR,ETV7,GJA1,STMN1,SLC9A3R1,VCAM1,TNFRSF1B,NFKBIA,MMP2,CASP3,HIF1A,IGF2,MYC,IGF2R,PXN,KCNQ1,CDH1,GNAI2,NOS2,P2RX7,CD8A,CALD1,PTGS2,PRKCB,SAT1,FAS,MAPK11,BID,CASP1,CASP2,CASP7,ITGB2,APP,CXCR4,CTGF,ST14,CCNE1,PLAU,FOXO1,VTN,LPAR2,PTTG1,CXCL12,P2RY6,PLK1,ITPR1,APOA1,SPP1,MMP9,CDKN1A,DDIT3,SPI1,BCAR1,CD4,CDK1,EGR1,PLA2G4A,EIF2AK2,NDRG1,TNFSF10,NOL3,C5,BSG,DPP4,TBP,PDCD4,CYBB,JAG1,RAC2,CD36,NOTCH1,CCL4,CHEK2,CDK6,ATR,PTPRC,NEDD4L,TLR2,MKI67,IL15,INHBA,NFE2L2,STAT3,PTPN13,POLG,POSTN,CD44,CTSK,APAF1,ARG2,CSK,NPM1,MTCH1,ITGA4,LAMA4,RASA1,MDM2,PIK3R1,WNT5A,NPPC,IRF1,GDF9,MLL,BMP2,PRNP,REL,LDHA,HSD11B1,SOCS1,PYCARD,CTSD,MYD88,COMP,CD38,SCARB1,RNF34,CTSB,RORA,PLIN2,OGT,DFFB,SREBF1,CEL,IL22,FOLR1,F3,CELF2,VCAN,C4orf14,SERPINA1,COL18A1,MGLL,CLDN5,SOAT1,MMP7,SOCS3,IL4R,PLTP,ID2,FGF1,MMP1,CDKN2A,AHR,LGALS1,XRCC6,TSC1,C3,IGFBP2,HDAC5,MCL1,TTR,MAPK9,ATRX,GLS,FASN,FGFR3,FYN,SMAD7,IFT88,SDC1,BCL2A1,TRPC3,TNS1,SFTPA1,NCF2,ECE1,CPE,MERTK,FAAH,NRP1,TLR3,RNASEL,NR1H3,IL1R1,TRPV6,NCOA2,FGFR2,PERP,KLF6,CUX1,TNFRSF18,AHSG,GLUL,MMP13,MGP,CCL17,MAFB,HSPG2,LGALS3,CFH,HPSE,ANXA2,PKD2,GAB1,TFPI,PTPN6,P2RX4,ZYX,FTH1,SLC31A1,CSF1R,EEF1A1,ACSL4,IL16,HSPE1,LTA4H,CD274,C1QA,RPL18A,TRERF1,WNT4,IFIH1,GAS6,C3AR1,NCOA1,CHRNA7,TXNRD2,MTHFR,ITGAV,EHF,FIGF,GPRC5A,UNC5B,C4BPA,SNAI1,CCL1,TNFSF13B,PDE3B,LAMP2,SLC6A2,TCIRG1,RGN,MRE11A,TICAM1,SLC6A6,CD82,HSP90AB1,TYMS,MST1,SLURP1,F8,CXADR,CTSS,BCL6,BTK,SERPINI1,BPI,GFRA1,PIK3CD,SPRY2,SOX4,CD83,RBL2,IRF8,XRCC5,LAMC1,CADM1,MGMT,IKZF1,CCL19,POLB,DAPK1,DNASE1L3,TSC22D3,ALDH1A2,B2M,MSH6,CDC20,PDE1A,SCIN,MEOX2,MFGE8,CDH11,IRF4,TCF3,DKC1,MCM5,MX1,ARHGDIB,PRKCH,RAD54L,TRAF6,GLI3,PTMA,MGAT3,CDC45,CD79B,ICMT,MVP,MTUS1,EPB41L3,CABC1,NCF4,STK4,TRPV2,NME1,TERF1,PML,PRKDC,CTCF,RAD50,GZMA,ID3,CDC14A,TFDP1,LIG4,BRD2,TAP1,CD81,CCNG1,CD247,DIAPH1,DUSP5,ROBO1,SFRP1,SMAD6,NF1,TIAL1,REV3L,GMNN,PIAS1,NCOR1,MLF1,MAP3K8,ATG7,IFT57,TRA@,CDT1,TNFAIP3,AKAP1,IFNAR1,TRIM24,FCER1G,GIMAP5,TPX2,M6PR,CD200,BHLHE40,HEY1,PDE4B,SUZ12,RAD9A,TOP2A,TRAF5,CAMKK2,DYNLT1,TGS1,UNG,BARD1,ITGB5,CD3E,FANCL,ALOX5AP,TWIST1,POT1,KLF10,STAG1,RIN2,CD74,MYCBP2,ITGB4,TNIP2,SH3KBP1,ELAC2,SRGN,FES,POU2AF1,SEPP1,LY6E,ZBTB17,REV1,GAS2,UGCG,TSC22D1,ULK1,USP18,USP28,GSG2,AGPAT2,SGMS1,HDAC4,NR2C2,SMYD3,PTPRF,ING2,SMARCAL1,NUMB,MARCKSL1,TES,HNRNPU,ORC2L,RXFP1,FOXG1,KLF11,ZEB1,RUVBL1,PPAP2B,HEXIM1,LGMN,TNFRSF21,KDM3A,DNAJA3,PAQR3,CDCA8,IFI27,PPP2R5A,MID1,HSF2,SEMA5A,PLEKHO1,RAD51L1,RANBP9,FBLN1,T,KIF23,TASP1,TWIST2,HOPX,PTP4A1,CSTA,RRM1,CTSL2,DPYD,EPHB3,JDP2,SLC16A2,TUB,RNASEH1,NEK1,SULF1,SEC14L2,PPP2R4,ORC1L,GSTO1,SGPP1,SMC6,MAP3K2,MBD4,SYNGAP1,QKI,SAP30,SIRT4,PHLPP1,HYAL1,NAV3,PLSCR3,BASP1,PIK3IP1,PACS2,CXXC1,KIF4A,AMOT,TIMM44,OSGIN1,E4F1,SGK3,CNKSR1,CDC34,S100A6,SRPK2,PSAP,GULP1,SUPT5H,RBBP7,CD3D,ING3,HIPK3,COX10,RRM2,IP6K2,CAMKK1,AATF,NSMCE2,PDCD10,LAMA3,FHOD1,MTMR9,ELF3,EEF1A2,DDX47,CREG1,FLNB,RPAP3,ZBTB38,ITPR3,NELL1,RHOBTB2,CHD1L,ZNF217,RBM5,LSP1,LZTR1,SDPR,DYRK2,UNC5C,GPR56,EFEMP1,SMPD3,SESN1,WISP3,AKR1B10,UNC119,ASAH1,FAIM,RFXANK,SPTBN1,SCARB2,ITGB3BP,CABIN1,TTC6,HAUS1,COQ2,LPCAT3,ELOVL5,DPYSL3,ARHGEF6,ARL6IP1,PIK3C2A,VPS53,TXNDC17,IRG1,PDCD6,HECW1,IGHMBP2,STK39,SENP1,MAGI2,CIRBP,TIMD4,MAP3K14,MYCT1,FZD8,PUS10,DUSP26,DNAJC12,TNIP1,LCMT1,PRIM1,ENOPH1,RPL18,FLII,P2RY13,HDAC10,LRIG3,EFHC1,IQCG,C2orf49,LITAF,C1QB,LYRM1,SUPV3L1,SPATA17,Wdr35l,TRIM69,LAPTM5,FAM129A,PRR5,RAB36,SRBD1,C7orf27,RABL3,TBC1D19	3.58E-30

up	F2,SRC,NPY,KNG1,MAPK8,TFF3,IL6,MME,SLC4A1,CAPN2,AQP3,ATP2B4,PTGDS,SELP,RB1,MTOR,TF,CAV1,CAMK4,HSP90B1,LPAR3,TACR1,AQP5,HSPA5,DUSP6,COL2A1,BDNF,ALOX5,NTF3,DUSP1,CSRP2,NR4A3,UTS2,INSR,IAPP,ADORA1,ID1,RGS4,PPARD,PTGS1,ANK2,FSHR,NRG1,PTH1R,ADORA2A,CNR1,CA9,GADD45B,SKP2,NCOA3,APOD,CYP7A1,MMP1,TGFB3,XBP1,PRDX6,PGGT1B,GJB1,LTF,PLCD1,ACE,PRLR,ENPP2,S1PR5,MSI1,STK11,GNAI1,LYZ,SFTPA1,ADA,ACTG2,MUC1,GNMT,GCH1,FABP3,KCNK1,OXTR,MMP12,NUCB2,SFRP4,ASPH,EEF1A1,BMPR2,GADD45G,PRKAR2B,HOMER2,ADSL,RCAN1,ENPEP,PDCD1,PRDX4,BECN1,TEC,RHOB,XPO1,UCHL1,PHB,GAB2,GFI1B,MOG,MYCN,MKL1,CSF3R,AMD1,STK25,TNFRSF6B,HDGFRP3,PEMT,PTN,MITF,PLXNA1,AMH,GREM1,SMARCD3,RAB3B,FKBP5,PLK3,NID1,NOV,PAEP,SNCA,SILV,NFIL3,MCM6,ETV4,DAG1,SOX7,CACNA1G,BHLHA15,P2RX5,HPX,EPS8,CREB3L2,LRRC3B,ASNS,SUFU,FGF19,POLR2G,RDX,PELP1,POLH,NDEL1,MFI2,NRCAM,UAP1,WNT5B,SH2D2A,GPR87,C8orf4,DEFB1,MYST4,NPDC1,TNN,NDC80,PRDM16,CCR4,RCAN2,SEMA3C,CACNA2D2,CRKL,PKP2,QSOX1,NELF,NRG2,APLP2,PSME3,MCF2,CDK8,COL8A1,PPP2R3A,RRBP1,POU6F1,FZD5,IL1RL1,TPD52,NUTF2,NEK8,PES1,CTHRC1,CREB3,ZNF384,LTBP3,BAALC,BCAT1,MCF2L,Kng1,ALDH18A1,KRT20,PSPH,NARS,PLXDC1	2.1862E-07

Proliferation	down	CEBPB,EGFR,ETV7,GJA1,STMN1,SLC9A3R1,VCAM1,TNFRSF1B,NFKBIA,MMP2,CASP3,CTTN,HIF1A,IGF2,MYC,IGF2R,PXN,KCNQ1,SLC9A3R2,CDH1,GNAI2,NOS2,P2RX7,CD8A,CALD1,PTGS2,PRKCB,SAT1,FAS,MAPK11,BID,CASP1,ITGB2,APP,CXCR4,CTGF,CCNE1,PLAU,FOXO1,VTN,LPAR2,PTTG1,CXCL12,P2RY6,PLK1,ITPR1,APOA1,SPP1,MMP9,CDKN1A,DDIT3,SPI1,BCAR1,CD4,CDK1,EGR1,PLA2G4A,EIF2AK2,CCL20,CCR2,NDRG1,TNFSF10,C5,BSG,DPP4,TBP,PDCD4,CYBB,JAG1,RAC2,CD36,NOTCH1,CCL4,CHEK2,CDK6,ATR,PTPRC,TLR2,MKI67,IL15,INHBA,NFE2L2,STAT3,PTPN13,POSTN,CD44,CTSK,ARG2,CSK,NPM1,ITGA4,LAMA4,RASA1,MDM2,PRKD2,PIK3R1,WNT5A,NPPC,IRF1,GDF9,MLL,BMP2,PRNP,REL,LDHA,HSD11B1,SOCS1,PYCARD,CTSD,MYD88,COMP,CD38,SCARB1,RNF34,CTSB,RORA,SREBF1,CEL,IL22,FOLR1,F3,CELF2,VCAN,SERPINA1,COL18A1,MMP7,CELF1,SOCS3,IL4R,ID2,FGF1,FRK,MMP1,CDKN2A,AHR,LGALS1,XRCC6,TSC1,C3,IGFBP2,MCL1,TTR,MAPK9,GLS,FASN,FGFR3,FYN,SMAD7,IFT88,SDC1,BCL2A1,TRPC3,ADC,SFTPA1,FBP1,ECE1,MERTK,FAAH,NRP1,TLR3,RNASEL,NR1H3,IL1R1,MAPKAPK2,TRPV6,NCOA2,FGFR2,PERP,KLF6,CUX1,PFKFB3,TNFRSF18,GLUL,MMP13,MGP,IPO13,MAFB,HSPG2,LGALS3,CFH,HPSE,ANXA2,PDGFA,PKD2,GAB1,TFPI,PTPN6,ZYX,FTH1,CSF1R,EEF1A1,ACSL4,IL16,HSPE1,LTA4H,CD274,C1QA,TRERF1,NPR3,WNT4,IFIH1,GAS6,NCOA1,ADAM33,CHRNA7,LIFR,TLR7,MTHFR,ITGAV,EHF,FIGF,GPRC5A,ADSL,EDN2,UNC5B,C4BPA,SNAI1,CCL1,TNFSF13B,TCIRG1,RGN,OSMR,MRE11A,OGN,CD82,TYMS,MST1,SLURP1,F8,CXADR,CTSS,BCL6,BTK,DOCK2,CD164,BPI,GFRA1,PIK3CD,SPRY2,AGRN,ANXA8L2,SOX4,CD83,RBL2,IRF8,IL2RG,BARX2,XRCC5,LAMC1,CADM1,MGMT,IKZF1,SLA,CCL19,TSC22D3,CNTN1,B2M,CDC20,PRSS21,PDE1A,SCIN,FZR1,MEOX2,MFGE8,CDH11,IRF4,TCF3,DKC1,RGS1,PRKCH,RAD54L,TRAF6,GLI3,PTMA,SOX5,CDC45,ALCAM,ICMT,MTUS1,EPB41L3,STK4,TRPV2,FKBP4,NME1,TERF1,PML,PRKDC,CTCF,RAD50,WFDC1,GZMA,ID3,HOXB3,TFDP1,LIG4,BRD2,HELLS,TAP1,CD81,CCNG1,CD247,Cr1l,DIAPH1,DUSP5,ROBO1,SFRP1,SMAD6,ID4,RBM15,NF1,F2RL2,TIAL1,TMPRSS2,GMNN,PIAS1,NCOR1,PTP4A3,HHEX,DPH1,IRF7,MLF1,SMARCA1,TRA@,CDT1,IFNAR1,FCER1G,TPX2,BHLHE40,HEY1,PDE4B,SUZ12,APCDD1,RAD9A,TOP2A,TRAF5,IL20RA,UNG,SH3GL1,LGR4,BARD1,CD3E,FANCL,ALOX5AP,TWIST1,POT1,KLF10,CD74,MYCBP2,ITGB4,ELAC2,FES,FOXE1,POU2AF1,BCAR3,MTAP,LY6E,ZBTB17,CDK10,GAS2,HMGB2,TSC22D1,USP28,AGPAT2,CSF2RA,RAD51C,NOLC1,HDAC4,NR2C2,SMYD3,CUEDC2,PTPRF,ING2,NUMB,MARCKSL1,DAB1,JARID2,ORC2L,RXFP1,FOXG1,KLF11,ZEB1,PTBP2,RUVBL1,PPAP2B,ADAM9,HEXIM1,KPNA2,LGMN,FABP7,TNFRSF21,RAP1GAP,RNASEN,DNAJA3,ATP6V0C,PAQR3,CDCA8,SKP1,LIN9,MID1,NPC1,PLEKHO1,CENPH,FBLN1,SDC3,SPAG9,T,CASC1,TASP1,UPF2,TWIST2,HOPX,PTP4A1,RRM1,DBF4,EPHB3,JDP2,SLC16A2,SULF1,SEC14L2,KDM2B,ORC1L,SCPEP1,RAD1,ARID5B,TGIF1,SMC6,ANAPC2,EXOC3,QKI,SAP30,MOBKL1A,SLBP,PHLPP1,HYAL1,EGFL7,UNC45B,PIK3IP1,CXXC1,DDX20,AMOT,TIMM44,WSB1,OSGIN1,E4F1,SGK3,CNKSR1,ILF3,GPR39,CLASP2,CDC34,S100A6,SRPK2,PSAP,DYNC1H1,LFNG,MT3,SORBS1,TSPAN1,HIPK3,IGLL1,RRM2,LCP1,AATF,COIL,DPYSL2,PDCD10,HOXB8,LAMA3,ITGB1BP1,ELF3,CREG1,CAPRIN1,ITPR3,IRAK4,L3MBTL2,NELL1,RHOBTB2,ZNF217,CALCOCO2,RBM5,FERMT1,BAMBI,EFEMP1,BTN1A1,TCOF1,SMPD3,RSL1D1,DECR1,ARNTL2,UTP3,WISP3,AKR1B10,UNC119,DHX30,MED23,CHRDL1,BCOR,FBXW8,ITGB3BP,VPS36,ATAD2,FIGNL1,RNF128,DNER,CAD,DPYSL3,PIK3C2A,OASL,OSCP1,PHC1,SENP1,RASGRP3,MAGI2,PPP2CA,CIRBP,SLC7A6,FBXO6,SH3BP2,CALM1,CSRNP1,PRIM1,RGS18,LGR5,LRIG3,DUSP11,PDS5A,ODZ1,IQCG,RASGEF1A,WDR3,LYRM1,CDCA4,KIAA1524,AK4,CHERP,PRR5,RPL15,TMEM132A,MACC1,RABL3	5.68E-21

	up	F2,SRC,NPY,ATP2B2,KNG1,MAPK8,TFF3,IL6,MME,CA2,CAPN2,AQP3,ATP2B4,PTGDS,SELP,RB1,MTOR,TF,CAV1,CAMK4,HSP90B1,TACR1,AQP5,HSPA5,DUSP6,COL2A1,BDNF,ALOX5,NTF3,DUSP1,RASD1,CSRP2,NR4A3,UTS2,INSR,IAPP,STX1A,ADORA1,ID1,PPARD,PTGS1,ANK2,FSHR,NRG1,PTH1R,HOMER1,ADORA2A,CNR1,CA9,GADD45B,SKP2,NCOA3,APOD,RARRES2,MMP1,TGFB3,XBP1,PGGT1B,GJB1,LTF,PLCD1,ACE,PRLR,S1PR5,SI,MSI1,STK11,GNAI1,ERP44,LYZ,SFTPA1,ADA,ACTG2,MUC1,FABP3,OXTR,MMP12,SFRP4,CETP,EEF1A1,BMPR2,CYP17A1,GADD45G,PRKAR2B,HDLBP,ENPEP,LTC4S,PDCD1,PRDX4,BECN1,TEC,RHOB,UCHL1,PHB,GAB2,GFI1B,MYCN,STEAP3,MKL1,CSF3R,AMD1,TNFRSF6B,PTN,MITF,AMH,GREM1,SMARCD3,PLK3,NID1,PAEP,SNCA,ETV4,DAG1,SOX7,NHLH2,BHLHA15,P2RX5,EPS8,ASNS,FGF19,LMAN1,RDX,NDEL1,MFI2,WDFY2,UAP1,WNT5B,DEFB1,MYST4,NPDC1,TNN,PRDM16,FHL3,NRG2,CRMP1,APLP2,MCF2,CDK8,NRXN1,SEC1,MIP,POU6F1,FZD5,IL1RL1,APBB1IP,CNTN2,DNAJB9,CTHRC1,ZRANB1,MRAS,EMB,ZNF384,RPL10,NAV2,BHLHE23,BAALC,TRIM2,TRIM3,HEXB,DNAJB11,LHX9,MUC5B,C1orf38,KRT20,TFCP2L1,KRT15,DPYSL4,SLC35B2,MTF2	1.2064E-06
Differentiation	down	CEBPB,EGFR,ETV7,GJA1,STMN1,VCAM1,TNFRSF1B,NFKBIA,MMP2,CASP3,CTTN,HIF1A,IGF2,MYC,IGF2R,PXN,KCNQ1,CDH1,GNAI2,NOS2,P2RX7,CD8A,CALD1,PTGS2,PRKCB,FAS,MAPK11,CASP1,ITGB2,APP,CXCR4,CTGF,ST14,CCNE1,PLAU,FOXO1,VTN,PTTG1,CXCL12,P2RY6,ITPR1,APOA1,SPP1,MMP9,CDKN1A,DDIT3,SPI1,BCAR1,CD4,CDK1,EGR1,PLA2G4A,EIF2AK2,CCR2,NDRG1,TNFSF10,C5,BSG,DPP4,TBP,PDCD4,CYBB,JAG1,RAC2,CD36,NOTCH1,CCL4,CHEK2,CDK6,ATR,PTPRC,TLR2,IL15,INHBA,NFE2L2,STAT3,PTPN13,POSTN,CD44,CTSK,CSK,NPM1,ITGA4,LAMA4,RASA1,MDM2,PIK3R1,WNT5A,NPPC,IRF1,GDF9,MLL,BMP2,PRNP,REL,HSD11B1,SOCS1,CTSD,MYD88,COMP,CD38,ANTXR1,SCARB1,CTSB,RORA,PLIN2,DFFB,SREBF1,IL22,F3,CELF2,VCAN,SERPINA1,COL18A1,MMP7,SOCS3,IL4R,PLTP,ID2,FGF1,FRK,MMP1,CDKN2A,AHR,LGALS1,TSC1,C3,IGFBP2,HDAC5,LY96,MCL1,MAPK9,DMBT1,ATRX,FASN,FGFR3,FYN,SMAD7,IFT88,SDC1,BCL2A1,TRPC3,ADC,LIPA,SFTPA1,FBP1,ECE1,FAAH,NRP1,TLR3,RNASEL,STOM,NR1H3,MAPKAPK2,TRPV6,NCOA2,FGFR2,KLF6,CUX1,TNFRSF18,AHSG,GLUL,MMP13,MGP,IPO13,MAFB,HSPG2,LGALS3,CFH,HPSE,ANXA2,PDGFA,PKD2,GAB1,PTPN6,P2RX4,ZYX,FTH1,SLC31A1,CSF1R,EEF1A1,IL16,HSPE1,PNRC1,CD274,C1QA,TRERF1,NPR3,WNT4,GAS6,C3AR1,NCOA1,LIFR,TLR7,ITGAV,EHF,FIGF,GPRC5A,EDN2,GLA,SNAI1,CCL1,TNFSF13B,PDE3B,SLC6A2,RGN,OSMR,TICAM1,OGN,CD82,HSP90AB1,WDR5,MST1,CETN3,SLURP1,F8,CXADR,CTSS,BCL6,BTK,CD164,GFRA1,PIK3CD,SPRY2,AGRN,ANXA8L2,SOX4,CD83,RBL2,IRF8,IL2RG,BARX2,XRCC5,LAMC1,CADM1,MGMT,IKZF1,SLA,CCL19,POLB,DAPK1,TSC22D3,ALDH1A2,CNTN1,B2M,MSH6,CDC20,PRSS21,SCIN,FZR1,MEOX2,CDH11,IRF4,TCF3,RGS1,PRKCH,TRAF6,GLI3,PTMA,MGAT3,SOX5,LCP2,CD79B,ALCAM,MVP,DPCD,MTUS1,FKBP4,NME1,TERF1,PML,PRKDC,CTCF,ID3,HOXB3,TFDP1,BRD2,CD81,CD247,DUSP5,SFRP1,SMAD6,ID4,RBM15,NF1,GMNN,NCOR1,HHEX,IRF7,MLF1,MAP3K8,LIMK2,SMARCA1,TRA@,CDT1,TRIM24,GIMAP5,CD200,BHLHE40,HEY1,SUZ12,DYNLT1,LGR4,CD3E,TWIST1,KLF10,ZNF521,MYCBP2,ITGB4,SH3KBP1,FES,FOXE1,POU2AF1,NBL1,MTAP,LY6E,ZBTB17,CDK10,CAMK2B,GAS2,UGCG,HMGB2,TSC22D1,ULK1,SPAG6,USP18,ST6GAL1,GSG2,CSF2RA,HDAC4,NR2C2,PTPRF,NUMB,DAB1,JARID2,ALDH1A1,FOXG1,KLF11,ZEB1,PTBP2,HEXIM1,KPNA2,FABP7,TNFRSF21,RAP1GAP,KDM3A,PREX1,RNASEN,DNAJA3,PAQR3,IFI27,LIN9,MID1,NPC1,PALB2,HSF2,PLEKHO1,RANBP9,FBLN1,SDC3,T,RHOQ,TLK1,TWIST2,HOPX,KDM6A,CTSL2,ZEB2,ELP2,UNC50,JDP2,SLC16A2,NANOS2,SLC23A2,CDON,ARID5B,TGIF1,QKI,SAP30,NAV3,CXXC1,ATN1,DDX20,OSGIN1,CHD4,SGK3,NET1,S100A6,PSAP,PORCN,LFNG,CTDSPL,LTBP1,MT3,SLC26A8,TSPAN1,HIPK3,IGLL1,DPYSL2,HOXB8,LAMA3,ITGB1BP1,ELF3,TCF7L1,CKB,CREG1,ASAP2,FLNB,SAMSN1,ITPR3,IRAK4,NELL1,PPHLN1,ZNF217,PLB1,GNAL,SMPD3,RNF111,PGD,SYNE2,UNC119,ASAH1,PGS1,FAIM,CHRDL1,SLC38A1,FIGNL1,RNF128,DNER,CEBPZ,ZNF277,DPYSL3,ARL6IP1,PHC1,HECW1,LPAR4,PLEKHB1,HEBP1,PPP2CA,RNPEP,SH3BP2,CLSTN1,MAP3K14,TPPP,SHANK2,ST3GAL5,ATXN10,RGS18,STRN,SPEN,RALGPS1,MBNL3,MYO9A,TBC1D2,IQCG,PITRM1,SFXN3,MAFK,ZMYND8,ZNF503	1.84E-14
