# Supplementary material for: Recrudescence Mechanisms and Gene Expression Profile of the Reproductive Tracts from Chickens during the Molting Period
Source: PLoS One. 2013 Oct 1;8(10):e76784. doi: 10.1371/journal.pone.0076784 (PMC3788108; doi:10.1371/journal.pone.0076784)
Supplement: Table S2 — Functional categorization of genes changed in the magnum between day 6 and day 12 during the molting period. (PDF) [file pone.0076784.s002.pdf]

Table S2. Functional categorization of genes changed in the magnum between day 6 and day 12 during the molting period.

Category	Day 6 vs. Day 12	Molecules	p-value
Apoptosis	up	FGF2,HSPB1,VTN,TF,SPP1,MMP9,COMP,FOLR1,APOD,MGLL,MMP7,MMP1,PGGT1B,GJB1,LGALS1,PRLR,TRPC3,SMOX,LYZ,AHSG,MMP13,CCL17,LGALS3,HPGD,CHRNA7,HOMER2,EHF,SOX4,CITED2,STEAP3,TNFRSF6B,PTN,MGAT3,CDC14A,A2M,ROBO1,PIAS1,NF2,TRIM24,FGF13,HBA1,CREB3L2,DKK3,SGPP1,CXXC1,SERPINB3,PSPH	0.000155333
	down	FHL2,MYLK,CDH2,CALD1,CD55,CAST,PKHD1,VIM,SLC9A2,TNNT2,CXCL12,PLA2G4A,EDNRA,CD36,PTPRC,CTSK,DCN,GDF9,SCARB1,NCAM1,CEL,FOLR1,VCAN,CSNK1E,EDNRB,FGFR3,TGFB2,DFFA,MMP13,COL3A1,KCNB1,ANXA2,GLRX,AKAP12,SNAI2,CCL1,PDE3B,SPARC,SDC2,SERPINB5,XPO1,RELN,GFRA1,S100A11,GLI3,CCNA1,NR0B1,FKBP5,FBXW7,MYH10,HERPUD1,RGS5,CD74,DCX,EPHA3,SOSTDC1,CDCA8,HSF2,TCF21,SLC6A1,TUB,GSTO1,NAV3,CD3D,FAIM2,SYNE1,AMIGO2,UNC119,GZMK,LITAF	4.79E-04
Proliferation	up	FGF2,HSPB1,VTN,TF,SPP1,MMP9,COMP,SLC46A1,FOLR1,APOD,MMP7,MMP1,PGGT1B,GJB1,LGALS1,PRLR,TRPC3,LYZ,FBP1,MMP13,LGALS3,HPGD,CHRNA7,HOMER2,EHF,SOX4,CITED2,TNFRSF6B,FZD9,PTN,A2M,ROBO1,PIAS1,NF2,PBK,HPX,CREB3L2,PA2G4,DKK3,NELF,CXXC1,ACPP,BCAT1,KRT20,PSPH,KRT23,TMEM132A	0.000293125
	down	FHL2,MYLK,CDH2,CALD1,CD55,CAST,PKHD1,VIM,CXCL12,PLA2G4A,EDNRA,CD36,PTPRC,CTSK,DCN,GDF9,SCARB1,NCAM1,CEL,FOLR1,VCAN,EDNRB,FGFR3,TGFB2,NPY5R,DFFA,MMP13,COL3A1,KCNB1,ANXA2,GLRX,PDCD1LG2,AKAP12,SNAI2,CCL1,SPARC,DIO3,SDC2,SERPINB5,XPO1,RELN,GFRA1,BARX2,S100A11,GLI3,CCNA1,WFDC1,NR0B1,FKBP5,FBXW7,MYH10,CD74,DCX,EPHA3,SOSTDC1,PIWIL1,CDCA8,TCF21,CASC1,SLC6A1,RTN4R,CLASP2,MT3,FBLN2,POU6F1,IL1RL1,HOXA3,GRIK1,SMOC2,UNC119,COL1A2,LGR5,HOXA6,RASGEF1A,MUSTN1	4.89E-05
Differentiation	up	FGF2,HSPB1,VTN,TF,SPP1,MMP9,COMP,APOD,MMP7,MMP1,PGGT1B,GJB1,LGALS1,PRLR,TRPC3,SMOX,LYZ,FBP1,AHSG,MMP13,LGALS3,HPGD,EHF,SOX4,CITED2,STEAP3,TNFRSF6B,FZD9,PTN,MGAT3,NF2,TRIM24,PBK,PA2G4,DKK3,CXXC1,PLB1,HSBP1,MUC5B,SHANK2,KRT20,HBA2	0.000558537
	down	FHL2,MYLK,CDH2,CALD1,CAST,PKHD1,VIM,SLC9A2,TNNT2,CXCL12,PLA2G4A,EDNRA,CD36,PTPRC,CTSK,DCN,GDF9,SCARB1,NCAM1,VCAN,CSNK1E,EDNRB,FGFR3,TGFB2,LIPA,MMP13,ANXA2,GLRX,AKAP12,SNAI2,CCL1,PDE3B,SPARC,DIO3,SDC2,SERPINB5,RELN,GFRA1,BARX2,S100A11,GLI3,CCNA1,SRD5A2,NR0B1,FBXW7,ZNF521,DCX,EPHA3,SPAG6,DSCAM,HSF2,TCF21,RTN4R,NAV3,MT3,FBLN2,POU6F1,IL1RL1,HOXA3,NPTX2,OLFM1,SYNE1,SMOC2,EMB,PDZRN3,UNC119,LPAR4,TPPP,HOXA6,HOXC9,MUSTN1	6.18E-06
