# Supplementary material for: Recrudescence Mechanisms and Gene Expression Profile of the Reproductive Tracts from Chickens during the Molting Period
Source: PLoS One. 2013 Oct 1;8(10):e76784. doi: 10.1371/journal.pone.0076784 (PMC3788108; doi:10.1371/journal.pone.0076784)
Supplement: Table S3 — Functional categorization of genes changed in the magnum between day 12 and day 20 during the molting period. (PDF) [file pone.0076784.s003.pdf]

Table S3. Functional categorization of genes changed in the magnum between day 12 and day 20 during the molting period.

Category	Day 12 vs. Day 20	Molecules	p-value
Apoptosis	up	CALCA,PKHD1,SPP1,MMP9,EGR1,C5,DPP4,CYBB,FGF1,TTR,SDC1,SLC25A4,GDNF,TNFRSF18,MMP13,LGALS3,C4BPA,CCL1,PDCD1,ATF2,GFRA1,CCL19,DNASE1L3,FCER1G,KLF10,FOXG1,CDCA8,T,CSTA,SGPP1,CXXC1	5.39108E-06
	down	TNFRSF1B,NPY,ATP2B2,BMP7,CALD1,AQP3,GNA13,CD55,CAST,ATP2B4,AGTR1,PTGDS,RB1,FN1,MEF2A,DCN,PTGS1,ANK2,NR3C2,NRG1,PTH1R,CA9,GADD45B,FST,EDNRB,GJB1,ACE,ATRX,S1PR5,STK11,ATF6,LYZ,TP53INP1,SFTPA1,ACTG2,FABP3,RBPJ,ZBTB16,FGF10,COL3A1,CFH,SLC31A1,KCNK5,GADD45G,IFIH1,SNAI2,ENPEP,SPARC,FLT4,CCNG2,PER2,RELN,TOP2B,GSTT1,TNFRSF6B,PTN,CDC14A,FKBP5,PLK3,A2M,NID1,PAEP,MYH10,SNCA,HBA1,NFIL3,DLC1,DCX,SEPP1,EPHA3,CREB3L2,NEDD9,FBLN1,PTPRD,TCF21,NEK1,NDC80,SEMA3C,ZAK,WIF1,ITSN1,PARP4,RRM2,DYNLL1,PTPRS,STK38L,UBASH3A,LAMA2,SMAD9,SYNE1,SPTBN1,CCNI,GDF10,SERPINB3,ITPK1,NAA50,CHAC1	2.03E-06
Proliferation	up	CALCA,PKHD1,SPP1,MMP9,EGR1,CCL20,C5,DPP4,CYBB,FGF1,TTR,SDC1,GDNF,TNFRSF18,MMP13,LGALS3,C4BPA,CCL1,PDCD1,ATF2,GFRA1,CCL19,FCER1G,KLF10,FOXG1,CDCA8,T,CXXC1,GRIK1,NELL2,SENP5,KRT23,RPL15	6.14693E-07
	down	TNFRSF1B,NPY,SLC19A1,BMP7,CALD1,AQP3,GNA13,CD55,CAST,ATP2B4,AGTR1,PTGDS,RB1,FN1,MEF2A,DCN,PTGS1,ANK2,NR3C2,NRG1,PTH1R,CA9,GADD45B,FST,EDNRB,GJB1,ACE,S1PR5,STK11,ATF6,LYZ,TP53INP1,SFTPA1,ACTG2,FABP3,RBPJ,ZBTB16,FGF10,COL3A1,CFH,ASPH,GADD45G,NPR3,IFIH1,SNAI2,ENPEP,SPARC,FLT4,CCNG2,PER2,RELN,TNFRSF6B,PTN,ALCAM,FKBP5,PLK3,A2M,NID1,PAEP,MYH10,SNCA,NFIL3,DLC1,DCX,EPHA3,CREB3L2,CRTC1,MOV10,PIWIL1,NEDD9,MFI2,FBLN1,TCF21,MYST4,ENAH,ARRDC3,NDC80,SEMA3C,RFC1,ZAK,WIF1,SYNPO2,EGFL7,QSOX1,NELF,MCF2,FBLN2,RRBP1,RRM2,IL1RL1,STK38L,LAMA2,SMAD9,C1QTNF3,SCARA5,COL1A2,GDF10,SLC7A6,LAPTM4B,NAA50	8.60E-07
Differentiation	up	CALCA,PKHD1,SPP1,MMP9,EGR1,C5,DPP4,CYBB,FGF1,SDC1,GDNF,TNFRSF18,MMP13,LGALS3,CCL1,PDCD1,ATF2,GFRA1,CCL19,KLF10,FOXG1,T,CXXC1,NELL2	0.00183045
	down	TNFRSF1B,NPY,ATP2B2,BMP7,CALD1,AQP3,GNA13,CAST,ATP2B4,AGTR1,PTGDS,RB1,FN1,MEF2A,RASD1,DCN,PTGS1,ANK2,NR3C2,NRG1,PTH1R,CA9,GADD45B,FST,EDNRB,GJB1,ACE,ATRX,S1PR5,STK11,ATF6,LYZ,SFTPA1,ACTG2,SLC1A3,FABP3,RBPJ,ZBTB16,FGF10,CFH,SLC31A1,GADD45G,NPR3,SNAI2,ENPEP,SPARC,FLT4,VIPR2,CCNG2,PER2,RELN,TOP2B,TNFRSF6B,PTN,ALCAM,PLK3,NID1,PAEP,SNCA,DCX,EPHA3,NEDD9,MFI2,FBLN1,TCF21,MYST4,ARRDC3,WIF1,SYNPO2,MCF2,FBLN2,NRXN1,IL1RL1,PTPRS,OLFM1,STK38L,LAMA2,SMAD9,SYNE1,ERBB2IP,PDZRN3,TTC3,EBF3,GDF10,MUC5B,ARHGAP21,ZMYND11,COL14A1,HBA2	3.39E-06
