# Supplementary material for: Recrudescence Mechanisms and Gene Expression Profile of the Reproductive Tracts from Chickens during the Molting Period
Source: PLoS One. 2013 Oct 1;8(10):e76784. doi: 10.1371/journal.pone.0076784 (PMC3788108; doi:10.1371/journal.pone.0076784)
Supplement: Table S4 — Functional categorization of genes changed in the magnum between day 20 and day 25 during the molting period. (PDF) [file pone.0076784.s004.pdf]

Table S4. Functional categorization of genes changed in the magnum between day 20 and day 25 during the molting period.

Category	Day 20 vs. Day 25	Molecules	p-value
Apoptosis	up	ETV7,NPY,NFKBIA,KCNQ1,CD8A,BMP7,ABCB1,CALD1,SAT1,CAST,CASP7,AGTR1,MAP2K4,RB1,SGK1,CD36,ROCK2,BNIP3,PTGS1,ITGA4,NR3C2,SCARB1,TNC,CA9,F3,EZR,FST,CLDN5,EDNRB,ACE,TP53INP1,SFTPA1,TLR3,FGFR2,PDE5A,ZBTB16,FGF10,GLUL,HPSE,ACSL4,IL16,CP,GADD45G,STK17A,IFIH1,FIGF,ENPEP,FLT4,CCNG2,TIRAP,PER2,RELN,MKL1,MX1,TNFRSF6B,SQSTM1,MAL,NR0B1,CDC14A,FKBP5,PLK3,NCOR1,MYH10,DLC1,FABP4,TNIP2,SRGN,SEPP1,USP18,TACC1,NEDD9,HSF2,PTPRD,PTP4A1,SLC6A1,FILIP1L,DYNLL1,PTPRS,UBASH3A,LAMA2,SMAD9,UNC5C,SYNE1,SESN1,MAGI2,CIRBP,Tel11q,CHAC1	2.96313E-07
	down	F2,CA2,AQP3,HSPB1,PTGDS,TF,PLK1,CAMK4,HSP90B1,CDK1,TACR1,HSPA5,CCNA2,MKI67,STX1A,ADORA1,HYOU1,FSHR,MDK,ERBB4,CNR1,NCOA3,APOD,PGGT1B,GJB1,LTF,LGALS1,BIRC5,NR1H4,LYZ,ADA,BRCA1,FABP3,MMP13,LGALS3,PRKAR2B,HMMR,HOMER2,PRDX4,Sprr1a,TYMS,RRM2B,GFRA1,AURKA,BUB1,RACGAP1,CDC20,MYCN,STEAP3,PTN,CKS1B,CDC45,BUB1B,GREM1,A2M,NOV,PIAS1,PAEP,NEK2,DHCR24,TTK,TPX2,TOP2A,CKAP2,FANCL,RPS27L,E2F8,DCX,BHLHA15,LMNB1,SGOL1,FDPS,SGCG,KIF23,KIF14,PDIA3,DKK3,RCAN2,KIF4A,RRM2,MTMR9,KERA,LGALS2,DNAJB11,DEPDC1,SERPINB3,PSPH,CA6,ITPK1	7.70E-05
Proliferation	up	ETV7,NPY,NFKBIA,KCNQ1,CD8A,BMP7,ABCB1,CALD1,SAT1,CAST,AGTR1,MAP2K4,RB1,SGK1,CD36,ROCK2,PTGS1,ITGA4,NR3C2,SCARB1,TNC,CA9,F3,EZR,FST,EDNRB,ACE,TP53INP1,SFTPA1,TLR3,FGFR2,PDE5A,ZBTB16,FGF10,GLUL,HPSE,ACSL4,IL16,CP,GADD45G,IFIH1,FIGF,ENPEP,OSMR,FLT4,CCNG2,PER2,RELN,SLA,CNTN1,PRSS21,MKL1,RGS1,TNFRSF6B,ALCAM,NR0B1,FKBP5,PLK3,NCOR1,MYH10,DLC1,FABP4,CRTC1,NEDD9,MFI2,PTP4A1,SLC6A1,ARRDC3,SYNPO2,MCF2,MT3,FILIP1L,IL1RL1,PHF1,LAMA2,SMAD9,C1QTNF3,TESC,SCARA5,OASL,MAGI2,CIRBP,SLC7A6,MTMR2,CSRNP1,RASGEF1A,LAPTM4B	2.00976E-06
	down	F2,AQP3,HSPB1,PTGDS,TF,PLK1,CAMK4,HSP90B1,CDK1,TACR1,HSPA5,CCNA2,MKI67,ADORA1,FSHR,MDK,ERBB4,CNR1,NCOA3,APOD,PGGT1B,GJB1,LTF,LGALS1,BIRC5,SOCS2,NR1H4,LYZ,ADA,BRCA1,FABP3,KCNK1,MMP13,LGALS3,ASPH,PRKAR2B,HMMR,HOMER2,PRDX4,TYMS,GFRA1,AURKA,BUB1,RACGAP1,CDC20,MYCN,CCNB2,FZD9,PTN,CKS1B,CDC45,PLXNA1,GREM1,RAB3B,A2M,CCNB3,NOV,PIAS1,PAEP,NEK2,TTK,PBK,SREBF2,TPX2,TOP2A,CKAP2,FANCL,E2F8,DCX,BHLHA15,P2RX5,KIF11,HPX,TK1,SUFU,FDPS,DLGAP5,KPNA2,PA2G4,UAP1,CKS2,KIF14,DKK3,RCAN2,SRM,NELF,CDK8,NUSAP1,RRM2,ACPP,CTHRC1,BCAT1,KRT20,PSPH,SRMS,KRT23,TMEM132A	1.35E-06
Differentiation	up	ETV7,NPY,NFKBIA,KCNQ1,CD8A,BMP7,ABCB1,CALD1,CAST,AGTR1,MAP2K4,RB1,SGK1,CD36,ROCK2,PTGS1,ITGA4,NR3C2,SCARB1,TNC,CA9,F3,EZR,FST,EDNRB,ACE,SFTPA1,TLR3,FGFR2,PDE5A,ZBTB16,FGF10,GLUL,HPSE,IL16,CP,GADD45G,FIGF,ENPEP,OSMR,FLT4,VIPR2,CCNG2,PER2,RELN,SLA,CNTN1,PRSS21,MKL1,RGS1,TNFRSF6B,MAL,ALCAM,NR0B1,PLK3,NCOR1,FABP4,USP18,NEDD9,MFI2,HSF2,ARRDC3,SYNPO2,MCF2,MT3,IL1RL1,NPTX2,PTPRS,LAMA2,SMAD9,SYNE1,ERBB2IP,TESC,TTC3,SLC38A1,LPAR4	1.35451E-06
	down	F2,CA2,AQP3,HSPB1,PTGDS,TF,CAMK4,HSP90B1,CDK1,TACR1,HSPA5,CCNA2,STX1A,ADORA1,FSHR,MDK,ERBB4,CNR1,NCOA3,APOD,PGGT1B,GJB1,LTF,LGALS1,BIRC5,SOCS2,NR1H4,LYZ,ADA,BRCA1,FABP3,MMP13,LGALS3,PRKAR2B,HMMR,HDLBP,PRDX4,GFRA1,AURKA,RACGAP1,CDC20,MYCN,CCNB2,STEAP3,FZD9,PTN,BUB1B,GREM1,CPEB1,PAEP,PBK,SREBF2,DCX,BHLHA15,P2RX5,KPNA2,LMAN1,PA2G4,UAP1,PRC1,DKK3,SRM,CDK8,SEC1,CTHRC1,RPL10,HSBP1,DNAJB11,MUC5B,KRT20,KRT15,SRMS	1.10E-02
