# Supplementary material for: Recrudescence Mechanisms and Gene Expression Profile of the Reproductive Tracts from Chickens during the Molting Period
Source: PLoS One. 2013 Oct 1;8(10):e76784. doi: 10.1371/journal.pone.0076784 (PMC3788108; doi:10.1371/journal.pone.0076784)
Supplement: Table S5 — Functional categorization of genes changed in the magnum between day 25 and day 30 during the molting period. (PDF) [file pone.0076784.s005.pdf]

Table S5. Functional categorization of genes changed in the magnum between day 25 and day 30 during the molting period.

Category	Day 25 vs. Day 30	Molecules	p-value
Apoptosis	up	CDH2,PTTG1,PLK1,CDK1,CCNA2,CREB1,MDK,CEL,PLTP,FGF1,LGALS1,MMP3,FABP3,RIPK2,MMP13,LGALS3,HMMR,C4BPA,GFRA1,AURKA,BUB1,RACGAP1,CDC20,CKS1B,EDIL3,CCNA1,BUB1B,NOV,PIAS1,NEK2,DHCR24,TTK,TPX2,HERPUD1,FANCL,E2F8,ATXN3,SGOL1,FDPS,NUF2,KIF23,KIF14,KIF4A,PDCD6,DEPDC1	1.84464E-05
	down	NPY,CAPN2,PKHD1,TF,CD38,CA9,NCS1,PGGT1B,ACE,IL16,ENPEP,CCL19,TNFRSF6B,GZMA,CD3E,RHOG,GZMK,CHAC1	3.97E-03
Proliferation	up	CDH2,PTTG1,PLK1,CDK1,CCNA2,CREB1,MDK,CEL,FGF1,LGALS1,MMP3,FABP3,RIPK2,MMP13,LGALS3,HMMR,C4BPA,DIO3,GFRA1,AURKA,BUB1,RACGAP1,CDC20,CKS1B,PLXNA1,CCNA1,NOV,PIAS1,NEK2,TTK,PBK,TPX2,FANCL,E2F8,KIF11,FDPS,DLGAP5,KPNA2,NUF2,CKS2,KIF14,STIL,NUSAP1,ATOH8,MUSTN1,TMEM132A	1.41758E-05
	down	NPY,CAPN2,PKHD1,TF,CD38,CA9,PGGT1B,ACE,HDC,IL16,ENPEP,CCL19,TNFRSF6B,GZMA,CD3E,HPX,MFI2,WNT5B	5.30E-03
Differentiation	up	CDH2,PTTG1,CDK1,CCNA2,CREB1,MDK,PLTP,FGF1,LGALS1,MMP3,LIPA,FABP3,RIPK2,MMP13,LGALS3,HMMR,DIO3,GFRA1,AURKA,RACGAP1,CDC20,EDIL3,CCNA1,SRD5A2,BUB1B,PBK,ATXN3,KPNA2,LTBP1,RPL10,ATOH8,MUSTN1	0.0303413
	down	NPY,CAPN2,PKHD1,TF,CD38,CA9,PGGT1B,ACE,HDC,IL16,ENPEP,CCL19,TNFRSF6B,CD3E,MFI2,WNT5B	9.06E-03
