# Supplementary material for: Recrudescence Mechanisms and Gene Expression Profile of the Reproductive Tracts from Chickens during the Molting Period
Source: PLoS One. 2013 Oct 1;8(10):e76784. doi: 10.1371/journal.pone.0076784 (PMC3788108; doi:10.1371/journal.pone.0076784)
Supplement: Table S6 — Functional categorization of genes changed in the magnum between day 30 and day 35 during the molting period. (PDF) [file pone.0076784.s006.pdf]

Table S6. Functional categorization of genes changed in the magnum between day 30 and day 35 during the molting period.

Category	Day 30 vs. Day 35	Molecules	p-value
Apoptosis	up	FHL2,GJA1,STMN1,MYH11,VCAM1,MYLK,TNFRSF1B,NPY,NFKBIA,MMP2,IGF2,MME,CDH2,BCL2,IGF2R,CD8A,ABCB1,CALD1,PRKCB,SLIT3,SAT1,CD55,FAS,CAST,BID,CASP2,ATP2B4,ITGB2,PKHD1,VIM,CXCR4,FGF7,FOXO1,GATA3,LPAR2,PTTG1,CXCL12,P2RY6,GSN,APOA1,PALLD,SPI1,CD4,S1PR1,PLA2G4A,TNFRSF11B,SOD3,CYBB,EDNRA,RAC2,CD36,NOTCH1,DUSP1,CCL4,CDK6,ATR,PTPRC,NEDD4L,TLR2,MKI67,IL15,NFE2L2,ANXA1,PTPN13,POSTN,CD44,CTSK,NPM1,DCN,ITGA4,LAMA4,PIK3R1,WNT5A,NPPC,IRF1,GDF9,MLL,PRNP,IGFBP5,SOCS1,MDK,PYCARD,COMP,CD38,SCARB1,NCOA6,ADAMTSL1,TNC,RORA,CA9,NCAM1,CEL,IL22,FOLR1,F3,VCAN,SERPINA1,TGM2,PLTP,ID2,FGF1,CDKN2A,EDNRB,AHR,PCSK2,LGALS1,PLAT,HDAC5,TTR,GLS,CX3CR1,FASN,FGFR3,FYN,CD40LG,TRIO,SMAD7,TGFB2,BCL2A1,TNS1,PROX1,BRCA1,CSNK1D,ECE1,CPE,TLR3,TRPV6,FGFR2,KLF6,GLUL,MGP,MAFB,COL3A1,CD34,CFH,SFRP2,EPAS1,KCNB1,ANXA2,PKD2,GAB1,TFPI,KLK1,ZYX,CSF1R,EEF1A1,ACSL4,GLRX,IL16,C1QA,STK17A,WNT4,GAS6,PDGFC,C3AR1,MTHFR,FIGF,AKAP12,ITGA1,C4BPA,SNAI2,CCL1,TNFSF13B,HK1,PDE3B,ENPEP,HOXA9,SPARC,SERPINE2,SDC2,RARB,TYMS,SLURP1,CTSS,BTK,XPO1,SERPINI1,MECOM,RELN,GFRA1,PIK3CD,SPRY2,CD83,HOXA7,IRF8,DLL1,XRCC5,GSTT1,ZAP70,IGFBP7,CCL19,DAPK1,TSC22D3,ALDH1A2,CRY1,B2M,CDC20,CD9,PDE1A,SCIN,MEOX2,MFGE8,CDH11,IRF4,KLF2,MCM5,MX1,ARHGDIB,PRKCH,CCR7,TFPI2,S100A11,CDH13,GLI3,PTMA,CDC45,CD79B,CABC1,TRPV2,NME1,FBLN5,TERF1,RUNX1T1,ABCC1,CCNA1,RAD50,GZMA,NR0B1,BUB1B,RASSF5,MCM2,WNT7A,TAP1,SERPINH1,CD81,CD247,NFIA,SFRP1,RND3,GMNN,MLF1,MAP3K8,IFT57,MYH10,NEK2,TRA@,TNFAIP3,DACH1,IFNAR1,FCER1G,GIMAP5,TPX2,M6PR,HEY1,DLC1,PDE4B,SUZ12,TOP2A,TRAF5,DYNLT1,CKAP2,UNG,BARD1,ITGB5,CD3E,ALOX5AP,RASSF3,STAG1,RGS5,BMPR1B,CD74,DCX,ITGB4,SRGN,POU2AF1,SEPP1,LY6E,REV1,GAS2,UGCG,EPHA3,USP18,SOSTDC1,SGMS1,HDAC4,NR2C2,MARCKSL1,RXFP1,FOXG1,ZEB1,RUVBL1,PPAP2B,CDCA8,NEDD9,MID1,HSF2,SEMA5A,PLEKHO1,CDK2AP1,FBLN1,T,TCF21,KIF23,TASP1,TWIST2,HOPX,PTP4A1,CSTA,FZD1,SLC6A1,SLC16A2,TUB,NINJ1,DUT,SEC14L2,GSTO1,SMC6,WIF1,QKI,PHLPP1,NAV3,PLSCR3,KIF4A,OSGIN1,FZD4,SRPK2,HLF,GULP1,RBBP7,CD3D,RRM2,FGL1,LAMA3,MTMR9,DYNLL1,EEF1A2,CREG1,ZBTB38,ITPR3,DYRK3,SLC17A5,LUM,ZNF217,RBM5,LSP1,SDPR,DYRK2,UNC5C,EFEMP1,SMPD3,NPTN,AMIGO2,AKR1B10,UNC119,SCARB2,UACA,ITGB3BP,CABIN1,HAUS1,COQ2,DPYSL3,ARHGEF6,ARL6IP1,JAZF1,HECW1,STK39,MAGI2,TIMD4,GZMK,DUSP26,TNIP1,PRIM1,P2RY13,LRIG3,DFNA5,EFHC1,IQCG,C2orf49,LITAF,C1QB,SPATA17,Wdr35l,LAPTM5,FAM129A,FNDC1,Tel11q,RAB36,TBC1D19	1.57E-26
	down	FGF2,F2,CCK,CA2,CLCN3,BCL2L1,AVP,AQP3,HBEGF,TF,LPAR1,TRAP1,TACR1,AREG,ATP2A3,ALOX5,UTS2,STX1A,ARG2,PTH1R,HOMER1,SLC7A11,CNR1,APOD,AR,MMP1,LTF,MMP3,ENPP2,CYP19A1,SMOX,MSI1,GNAI1,SLC25A4,ATF3,FABP3,NLRP3,RIPK2,MMP13,LGALS3,PRKAR2B,HOMER2,RCAN1,Sprr1a,RHOB,RRM2B,BCR,NFYB,STEAP3,MKL1,TNFRSF6B,PTN,MGAT3,SERPINB2,CBFB,FDXR,GREM1,DCLRE1C,TUSC2,NOV,IFNAR1,FGF13,CAMKK2,SPDEF,BHLHA15,CREB3L2,ASNS,RALB,SGCG,POLH,NRCAM,CPD,DUSP4,KIF14,AGR2,EPRS,ADCY10,LGALS4,MTMR9,LGALS2,PDCD6,MIB2,PSPH,NARS,EAF2,ITPK1,LGALS12,CHAC1	1.07E-03

Proliferation	up	FHL2,GJA1,STMN1,MYH11,VCAM1,MYLK,TNFRSF1B,NPY,NFKBIA,MMP2,IGF2,MME,CDH2,BCL2,IGF2R,SLC9A3R2,CD8A,ABCB1,CALD1,PRKCB,SAT1,CD55,FAS,CAST,BID,ATP2B4,ITGB2,PKHD1,VIM,CXCR4,FGF7,FOXO1,GATA3,LPAR2,PTTG1,CXCL12,P2RY6,GSN,APOA1,SPI1,CD4,S1PR1,PLA2G4A,TNFRSF11B,SOD3,CYBB,EDNRA,RAC2,CD36,NOTCH1,DUSP1,CCL4,CDK6,ATR,PTPRC,TLR2,MKI67,IL15,NFE2L2,ANXA1,PTPN13,POSTN,CD44,CTSK,NPM1,DCN,ITGA4,LAMA4,PRKD2,PIK3R1,WNT5A,NPPC,IRF1,GDF9,MLL,PRNP,LDLR,IGFBP5,SOCS1,MDK,PYCARD,COMP,CD38,SCARB1,NCOA6,ADAMTSL1,TNC,RORA,CA9,NCAM1,ABCC4,CEL,IL22,FOLR1,F3,VCAN,SERPINA1,TGM2,CELF1,ID2,FGF1,CDKN2A,EDNRB,AHR,PCSK2,LGALS1,HDC,PLAT,TTR,GLS,CX3CR1,FASN,FGFR3,FYN,CD40LG,TRIO,SMAD7,TGFB2,BCL2A1,PROX1,BRCA1,ECE1,TLR3,TRPV6,FGFR2,KLF6,MMP12,PFKFB3,GLUL,MGP,MAFB,COL3A1,CD34,CFH,SFRP2,EPAS1,KCNB1,ANXA2,PDGFA,PKD2,GAB1,TFPI,KLK1,ZYX,CSF1R,EEF1A1,ACSL4,GLRX,IL16,C1QA,NPR3,WNT4,GAS6,PDGFC,ADAM33,MTHFR,FIGF,AKAP12,ITGA1,EDN2,C4BPA,SNAI2,CCL1,TNFSF13B,ENPEP,HOXA9,SPARC,SERPINE2,DIO3,OGN,SDC2,RARB,TYMS,SLURP1,CTSS,BTK,DOCK2,XPO1,MECOM,RELN,GFRA1,PIK3CD,SPRY2,AGRN,ANXA8L2,CD83,HOXA7,IRF8,IL2RG,DLL1,BARX2,XRCC5,LMO2,ZAP70,IGFBP7,SLA,CCL19,TSC22D3,CNTN1,B2M,CDC20,PRSS21,CD9,PDE1A,SCIN,MEOX2,MFGE8,CDH11,IRF4,KLF2,RGS1,PRKCH,CCR7,TFPI2,S100A11,CDH13,GLI3,PTMA,SOX5,CDC45,ALCAM,TRPV2,NME1,FBLN5,TERF1,RUNX1T1,CCNA1,RAD50,WFDC1,GZMA,NR0B1,HOXB3,KCNA4,HELLS,MCM2,WNT7A,TAP1,SERPINH1,CD81,CD247,SFRP1,ID4,RND3,F2RL2,GMNN,CXCL14,HHEX,IRF7,MLF1,MYH10,NEK2,SMARCA1,TRA@,DACH1,IFNAR1,FCER1G,MCM7,TPX2,HEY1,DLC1,PDE4B,MCM6,SUZ12,APCDD1,TOP2A,TRAF5,CKAP2,UNG,BARD1,CD3E,ALOX5AP,RASSF3,BMPR1B,CD74,DCX,ITGB4,POU2AF1,MTAP,LY6E,CDH4,GAS2,HMGB2,BCHE,EPHA3,SPRY1,CSF2RA,SOSTDC1,HDAC4,NR2C2,CRTC1,TK1,MARCKSL1,DAB1,RXFP1,FOXG1,DLGAP5,ZEB1,RUVBL1,PPAP2B,PIWIL1,CDCA8,NEDD9,LIN9,MID1,NPC1,PLEKHO1,CENPH,CDK2AP1,FBLN1,T,TCF21,CASC1,TASP1,TWIST2,HOPX,PTP4A1,DBF4,FZD1,SLC6A1,SLC16A2,ARRDC3,DUT,SEC14L2,SCPEP1,SMC6,WIF1,QKI,SYNPO2,ANTXR2,EMP1,SLBP,PHLPP1,RBM38,EGFL7,OSGIN1,FZD4,SRPK2,HLF,LFNG,MT3,FBLN2,NDRG4,IGLL1,POU6F1,RRM2,LCP1,IL1RL1,COIL,FGL1,DPYSL2,HOXA3,LAMA3,LIMA1,RARRES1,CREG1,ITPR3,LUM,ZNF217,RBM5,EFEMP1,BTN1A1,SMPD3,NPTN,TESC,SMOC2,ZNF423,AKR1B10,UNC119,SCARA5,CHRDL1,COL1A2,ITGB3BP,FIGNL1,DNER,DPYSL3,OASL,JAZF1,OSCP1,RASGRP3,MAGI2,SLC7A6,FBXO6,CALM1,CSRNP1,PRIM1,RGS18,LGR5,HOXA6,LRIG3,DUSP11,ODZ1,IQCG,RASGEF1A,PID1,RNF7,KIAA1524,MUSTN1	2.67E-27
	down	FGF2,F2,CCK,CLCN3,BCL2L1,AVP,AQP3,HBEGF,GJB6,TF,LPAR1,TRAP1,TACR1,AREG,ATP2A3,ALOX5,UTS2,ARG2,PTH1R,SLC7A11,CNR1,APOD,AR,MMP1,LTF,MMP3,ENPP2,CYP19A1,MSI1,GNAI1,ATF3,FABP3,KCNK1,RIPK2,MMP13,LGALS3,YARS,LEFTY2,PRKAR2B,HOMER2,RCAN1,RHOB,DDR2,BCR,NFYB,MKL1,TNFRSF6B,FZD9,PTN,SERPINB2,CBFB,PLXNA1,GREM1,VPS54,RAB3B,NOV,IFNAR1,SPDEF,CACNA1G,BHLHA15,P2RX5,HPX,CREB3L2,ASNS,RALB,POLH,CGN,NRCAM,DUSP4,KIF14,AGR2,STIM2,SRM,QSOX1,FLRT3,CDK8,RAB11FIP4,ACPP,LTBP3,BCAT1,MCF2L,WNK2,CUL2,ALDH18A1,PSPH,NARS,SRMS,EAF2,KRT23	1.53E-03

Differentiation	up	FHL2,GJA1,STMN1,MYH11,VCAM1,MYLK,TNFRSF1B,KCNJ2,NPY,NFKBIA,MMP2,IGF2,MME,CDH2,BCL2,IGF2R,CD8A,ABCB1,CALD1,PRKCB,FAS,CAST,ATP2B4,ITGB2,PKHD1,VIM,CXCR4,FGF7,FOXO1,GATA3,PTTG1,CXCL12,P2RY6,GSN,APOA1,PALLD,SPI1,CD4,S1PR1,PLA2G4A,TNFRSF11B,CYBB,EDNRA,RAC2,CD36,NOTCH1,DUSP1,CCL4,CDK6,ATR,PTPRC,TLR2,IL15,NFE2L2,ANXA1,PTPN13,POSTN,CD44,CTSK,NPM1,DCN,ITGA4,LAMA4,PIK3R1,WNT5A,NPPC,IRF1,GDF9,MLL,PRNP,IGFBP5,SOCS1,MDK,COMP,CD38,ANTXR1,SCARB1,NCOA6,ADAMTSL1,TNC,RORA,CA9,NCAM1,IL22,F3,VCAN,SERPINA1,TGM2,PLTP,ID2,FGF1,CDKN2A,EDNRB,AHR,PCSK2,LGALS1,HDC,PLAT,HDAC5,LY96,DMBT1,FASN,FGFR3,FYN,CD40LG,TRIO,SMAD7,TGFB2,BCL2A1,LIPA,PROX1,BRCA1,ECE1,TLR3,STOM,TRPV6,FGFR2,KLF6,MMP12,GLUL,MGP,MAFB,CD34,CFH,SFRP2,EPAS1,ANXA2,PDGFA,PKD2,GAB1,ZYX,CSF1R,EEF1A1,GLRX,IL16,C1QA,TPM1,NPR3,WNT4,GAS6,C3AR1,FIGF,AKAP12,EDN2,SNAI2,CCL1,TNFSF13B,PDE3B,ENPEP,HOXA9,SPARC,SERPINE2,DIO3,OGN,SDC2,RARB,CETN3,SLURP1,CTSS,BTK,MECOM,RELN,GFRA1,PIK3CD,SPRY2,AGRN,ANXA8L2,CD83,HOXA7,IRF8,IL2RG,DLL1,BARX2,XRCC5,LMO2,ZAP70,IGFBP7,SLA,CCL19,DAPK1,TSC22D3,ALDH1A2,CNTN1,B2M,CDC20,PRSS21,CD9,SCIN,MEOX2,CDH11,IRF4,KLF2,RGS1,PRKCH,CCR7,TFPI2,S100A11,CDH13,GLI3,PTMA,SOX5,LCP2,CD79B,ALCAM,DPCD,NME1,TERF1,RUNX1T1,ABCC1,CCNA1,NR0B1,BUB1B,HOXB3,KCNA4,CETN2,WNT7A,CD81,CD247,NFIA,SFRP1,ID4,GMNN,CXCL14,HHEX,IRF7,MLF1,MAP3K8,SMARCA1,TRA@,DACH1,GIMAP5,HEY1,SUZ12,DYNLT1,CD3E,BMPR1B,ZNF521,DCX,ITGB4,POU2AF1,MTAP,LY6E,CDH4,CAMK2B,GAS2,UGCG,HMGB2,EPHA3,SPAG6,USP18,SPRY1,ST6GAL1,CSF2RA,HDAC4,NR2C2,DAB1,ALDH1A1,FOXG1,ZEB1,DSCAM,PREX1,NEDD9,LIN9,MID1,NPC1,PALB2,HSF2,PLEKHO1,CDK2AP1,FBLN1,T,TCF21,TWIST2,HOPX,FZD1,ZEB2,SLC16A2,ARRDC3,SLC23A2,WIF1,QKI,SYNPO2,EMP1,NAV3,ATN1,OSGIN1,LFNG,CTDSPL,LTBP1,MT3,FBLN2,NDRG4,IGLL1,POU6F1,IL1RL1,APBB1IP,FGL1,DPYSL2,HOXA3,LAMA3,NPTX2,RARRES1,OLFM1,CKB,CREG1,SAMSN1,ITPR3,SLC17A5,ZNF217,CBY1,SMPD3,ERBB2IP,NPTN,TESC,SMOC2,EMB,PDZRN3,ZNF423,UNC119,CHRDL1,SLC38A1,FIGNL1,DNER,DPYSL3,ARL6IP1,HECW1,LPAR4,HEBP1,CLSTN1,TPPP,RGS18,RALGPS1,TAF1A,HOXA6,HOXC9,COL14A1,IQCG,GDPD5,PID1,MUSTN1,ZMYND8	2.47E-20
	down	FGF2,F2,CCK,CA2,CLCN3,BCL2L1,AVP,AQP3,HBEGF,TF,LPAR1,TRAP1,TACR1,AREG,ALOX5,RASD1,UTS2,STX1A,PTH1R,HOMER1,CNR1,APOD,AR,MMP1,LTF,MMP3,CYP19A1,SMOX,MSI1,GNAI1,ATF3,FABP3,RIPK2,MMP13,LGALS3,KALRN,ABCA3,LEFTY2,PRKAR2B,LTC4S,RHOB,BCR,STEAP3,MKL1,TNFRSF6B,FZD9,PTN,MGAT3,SERPINB2,CBFB,GREM1,DCLRE1C,VPS54,ZAR1,SPDEF,BHLHA15,P2RX5,ASNS,CGN,WDFY2,AGR2,EPRS,Akr1b7,SRM,FLRT3,ROS1,CDK8,PORCN,RAB11FIP4,ROD1,RPL10,SLC38A1,BHLHE23,SHANK2,TFCP2L1,KRT15,SRMS	5.90E-03
